# Co-creating Research Integrity Education Guidelines for Research Institutions

**DOI:** 10.1007/s11948-023-00444-2

**Published:** 2023-07-20

**Authors:** Krishma Labib, Natalie Evans, Daniel Pizzolato, Noémie Aubert Bonn, Guy Widdershoven, Lex Bouter, Teodora Konach, Miranda Langendam, Kris Dierickx, Joeri Tijdink

**Affiliations:** 1 Amsterdam University Medical Centers, Vrije Universiteit Amsterdam, Department of Ethics, Law and Humanities, Amsterdam Public Health Institute, De Boelelaan 1117, 1081HV Amsterdam, The Netherlands; 2 Centre for Biomedical Ethics and Law, Department of Public Health and Primary Care, University of Leuven, Kapucijnenvoer 35, Box 7001, 3000 Louvain, Belgium; 3 Department of Philosophy, Vrije Universiteit Amsterdam, De Boelelaan 1105, 1081 HV Amsterdam, The Netherlands; 4 Amsterdam University Medical Centers, Department of Biostatistics and Data Science, Amsterdam Public Health Institute, Vrije Universiteit Amsterdam, De Boelelaan 1117, 1081HV Amsterdam, The Netherlands; 5 Austrian Agency for Research Integrity, Landstraßer Hauptstraße 9, TOP 21, 1030 Vienna, Austria; 6 Amsterdam University Medical Centers, University of Amsterdam, Department of Biostatistics and Data Science, Amsterdam Public Health Institute, Meibergdreef 9, 1105 AZ Amsterdam, The Netherlands

**Keywords:** Education, Training, Research integrity, Responsible conduct of research, Guidelines, Institutions

## Abstract

To foster research integrity (RI), research institutions should develop a continuous RI education approach, addressing various target groups. To support institutions to achieve this, we developed RI education guidelines together with RI experts and research administrators, exploring similarities and differences in recommendations across target groups, as well as recommendations about RI education using approaches other than formal RI training. We used an iterative co-creative process. We conducted four half-day online co-creation workshops with 16 participants in total, which were informed by the RI education evidence-base. In the first two workshops, participants generated ideas for guidelines’ content, focusing on different target groups and various approaches to RI education. Based on this content we developed first drafts of the guidelines. Participants in the third and fourth workshop refined those drafts. We next organized a working group which further prioritized, reorganized, and optimized the content of the guidelines. We developed four guidelines on RI education focusing on (a) bachelor, master and PhD students; (b) post-doctorate and senior researchers; (c) other RI stakeholders; as well as (d) continuous RI education. Across guidelines, we recommend mandatory RI training; follow-up refresher training; informal discussions about RI; appropriate rewards and incentives for active participation in RI education; and evaluation of RI educational events. Our work provides experience-based co-created guidance to research institutions on what to consider when developing a successful RI education strategy. Each guideline is offered as a distinct, publicly available tool in our toolbox (www.sops4ri.eu/toolbox) which institutions can access, adapt and implement to meet their institution-specific RI education needs.

*Trial registration* https://osf.io/zej5b.

**Supplementary Information:**

The online version contains supplementary material available at 10.1007/s11948-023-00444-2.

## Background

Research integrity (RI) can be defined as doing research according to high professional, methodological and ethical standards (Boehme et al., 2016), and is crucial for producing trustworthy research findings. Fostering RI is the joint responsibility of multiple stakeholders (Bouter, 2018) because RI is influenced by various individual, institutional, and systemic factors. These include researchers’ personal character traits and ethical decision making skills (Tijdink et al., 2016), the departmental research culture (Haven et al., 2019; Joynson & Leyser, 2015), availability of responsible leadership (Pizzolato et al., 2022), and assessment criteria for funding, hiring and promotion (Aubert Bonn & Bouter, 2021; Titus et al., 2008). Since researchers and their behaviors are highly dependent on the infrastructures, procedures, support systems, and research environments present at their institution, research institutions, in particular, play an important role in fostering RI (Mejlgaard et al., 2020).

One of the core responsibilities of research institutions is to provide RI education and training (All European Academies, 2017; Mejlgaard et al., 2020), with some countries even having legal mandates for researchers or research institutions receiving public funding, such as the US (Kalichman, 2014). RI education is thought to shape knowledge, skills, and attitudes towards RI and thereby increase awareness about responsible research practices (RRPs) and questionable research practices (QRPs) (Kalichman & Plemmons, 2007; Labib et al., 2021b), and contribute to a better research culture (Kalichman, 2014). QRPs consist of practices that do not count as outright misconduct but can hamper the quality of research, such as selective reporting, hypothesizing after results are known, p-hacking, or poor supervision. The terms ‘education’ and ‘training’ are often used interchangeably and there are numerous ways to define them (Masadeh, 2012). In this paper, we use the term ‘RI education’ to refer to all approaches used to develop researchers’ cognitive and moral understanding of, and skills related to, RI. On the other hand, we say ‘training’ when addressing specific formal instructional events used for RI education (e.g. courses, workshops). Thus, we see RI training as an important aspect of RI education.

There is an increasing provision of RI trainings globally (Abdi et al., 2021a, 2021b; Evans et al., 2021; Kalichman, 2014; Mejlgaard et al., 2020), but these are typically developed without being part of a general overarching institutional RI education strategy (Kalichman, 2014; Kalichman & Plemmons, 2007), and as such there is a risk that trainings are experienced as one-off events which have little impact on participants’ long-term behavior (Barnes et al., 2006). Furthermore, most existing RI educational events target PhD students, even though research shows that diverse stakeholders also see the need for targeting other students (i.e., at the bachelor and master level), researchers across ranks, as well as other institutional stakeholders involved in research such as institutional leaders and RI policy makers (Labib et al., 2021a). Because of the diverse needs of various research stakeholders (Labib et al., 2021a), it might be that different educational strategies are required for different stakeholders (e.g., students as opposed to senior researchers). Moreover, it might be that RI training is not sufficient in providing adequate RI education, considering that RI education also takes place in informal ways, such as through supervision and socialization in the research process (Labib et al., 2021a).

It could be valuable and efficient for research institutions to develop an RI education strategy which includes educational approaches tailored to different target groups (including students, researchers and other institutional stakeholders) and allows for continuous RI education (Barnes et al., 2006; Labib et al., 2021a). RI education guidelines, entailing recommendations and best practices, can provide considerations for institutions on what to include in their institutional RI education strategy. By ‘guidelines’, we refer to documents containing guidance, and by ‘recommendations’ we refer to the specific items in the guidelines. To ensure that guidelines are sensitive to stakeholders’ actual RI education needs, they should be focused on practice and incorporate the perspectives and experiences of various research stakeholders. A co-creative approach to developing the guidelines, where stakeholders are not only consulted but also directly involved in the guideline development process, is helpful to achieve this (den Breejen et al., 2012; Labib et al., 2021b).

Together with various research stakeholders, we used an iterative co-creative methodology, which resulted in co-created guidelines on RI education for research institutions. In this paper, we describe the development of these guidelines and reflect on them by focusing on three questions: (1) Which recommendations are applicable across various RI education target groups?; (2) Are there any specific recommendations that are applicable to some target groups but not others?; and (3) What additional recommendations to research institutions (i.e. institutional officials and decision makers) are needed to increase awareness about RI in the institution, other than providing RI training?

## Methods

**Fig. 1:**
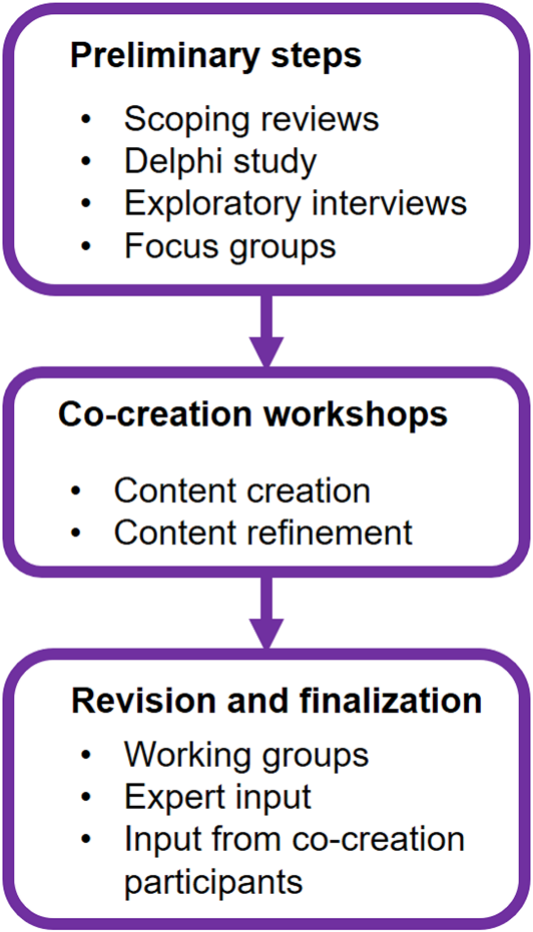
Guideline co-creation process. Since the preliminary steps have been discussed in detail elsewhere (Labib, Evans, et al., 2021; Labib et al., 2021a, 2021b, 2021c; Roje et al., 2021, 2022; Ščepanović et al., 2021; Sørensen et al., 2021), this manuscript elaborates more on the co-creation workshops and revision and finalization steps of the guideline development process

The guidelines presented in this paper are the result of a combination of iterative steps used to co-create guidelines on a number of distinct RI topics (Fig. 1). Here, we focus on the methods and results relating specifically to the guidelines on the topic of RI education. To obtain a first overview of potential considerations to include in these guidelines, we used insights from several previous empirical studies, which can be seen as preliminary steps in our guideline development process (Gaskell et al., 2019; Labib, Evans, et al., 2021; ; Ščepanović et al., 2021; Sørensen et al., 2021). We next conducted four co-creation workshops together with various stakeholders to develop the guidelines, and then formed a working group to further revise and operationalize the developed guidelines.

### Preliminary Steps

We identified available recommendations on the topic of RI education, as well as gaps, by: (1) performing scoping reviews on best practices for RI promotion (including RI education) (Ščepanović et al., 2021) and the factors for successful implementation of these (Roje et al., 2022); (2) conducting 23 interviews with RI experts (Roje et al., 2021); and (3) a Delphi consensus-study with 68 research policy makers and research leaders across Europe (Labib et al., 2021a, 2021b, 2021c). Informed by these studies, we then conducted 30 focus groups with researchers and other research stakeholders from different disciplines and countries in Europe (Labib et al., 2021a) to explore their perspectives and preferences regarding RI education. Based on the insights gained, we compiled a comprehensive list of possible recommendations for research institutions on RI education (Lechner et al., 2020), which were represented as ‘inspirations’ (elaborated on further below) and served as input for a set of co-creation workshops.

### Co-creation Workshops

We conducted four co-creation workshops to jointly develop the RI guidelines together with various research stakeholders. The workshop methods have been described in detail elsewhere (Labib et al., 2021b). The workshops included active involvement—rather than mere consultation—of stakeholders from the onset of the guideline development process (Labib et al., 2021b). We followed a co-creation workshop approach as elaborated on by Sanders and Stappers (2012), where stakeholders are engaged in creative workshops to jointly develop user-centered outputs. The workshops stimulated stakeholders to reflect on their experiences with RI education through the use of various interactive exercises making use of visual and textual stimuli to create ideas for guidelines, and then discuss these with others to build on each other’s ideas, prioritize ideas, and make joint conclusions (Labib et al., 2021b). The methods were aimed at incorporating the actual needs and perspectives of stakeholders (Sanders & Stappers, 2012), and can be considered particularly valuable for eliciting a broad range of ideas (Labib et al., 2021b).

#### Participants

We used a purposive recruitment strategy to identify and invite participants who were potential lead users of the guidelines (i.e., have a responsibility in their implementation). Participants included RI experts and research administrators representing different countries, professional roles, and genders. We aimed to recruit 4–6 participants per workshop to allow for in-depth discussions (Labib et al., 2021b). Other relevant stakeholders’ specific needs in relation to RI education—including those of junior researchers and PhD students—identified in the preliminary steps (e.g., focus groups), were fed into the co-creation workshops as preparatory material that workshop participants received before joining the workshops. To invite participants, we simultaneously (1) approached contacts from our networks via email, followed by snowballing, and (2) approached people listed in internal databases of RI experts (e.g., ENERI, https://eneri.eu/; EARMA, https://www.earma.org/). We recruited 16 participants in total trough this strategy (Table 1).

**Table 1:** Characteristics of participants

Characteristics	Number of participants
*Participating in each workshop*	
Workshop 1	4
Workshop 2	5
Workshop 3	4*
Workshop 4	5*
**Total**	**16**
*Gender*	
Female	10
Male	6
Stakeholder type**	
Research manager	6
Senior researcher	2
Research head	1
RI coordinator	6
Publisher	1
*Country*	
Belgium	2
Finland	1
Germany	1
Ireland	2
Italy	1
Lithuania	1
Netherlands	2
Spain	3
Sweden	1
Switzerland	1
UK	1
**Total number of countries**	**11**

*One of these participants had also contributed to the first or second workshop

**We categorized participants’ roles based on their job titles and positions ‘Research manager’ includes stakeholders with job titles such as ‘research manager’, ‘research support manager’, ‘graduate education officer’, ‘research integrity officer/manager’, and ‘assistant to ombudsperson’. Senior researcher includes researchers who are assistant, associate or full professors.’ ‘Research head’ refers to researchers with positions as department, faculty, or institution-wide leads (e.g., department heads, rectors). ‘RI coordinator’ includes those with job titles such as ‘RI coordinator’ or ‘ethics coordinator’, ‘research coordinator’, and ‘scientific coordinator’. ‘Publisher’ refers to participants primarily representing a research publisher

#### Workshop Set-up and Organization

**Fig. 2:**
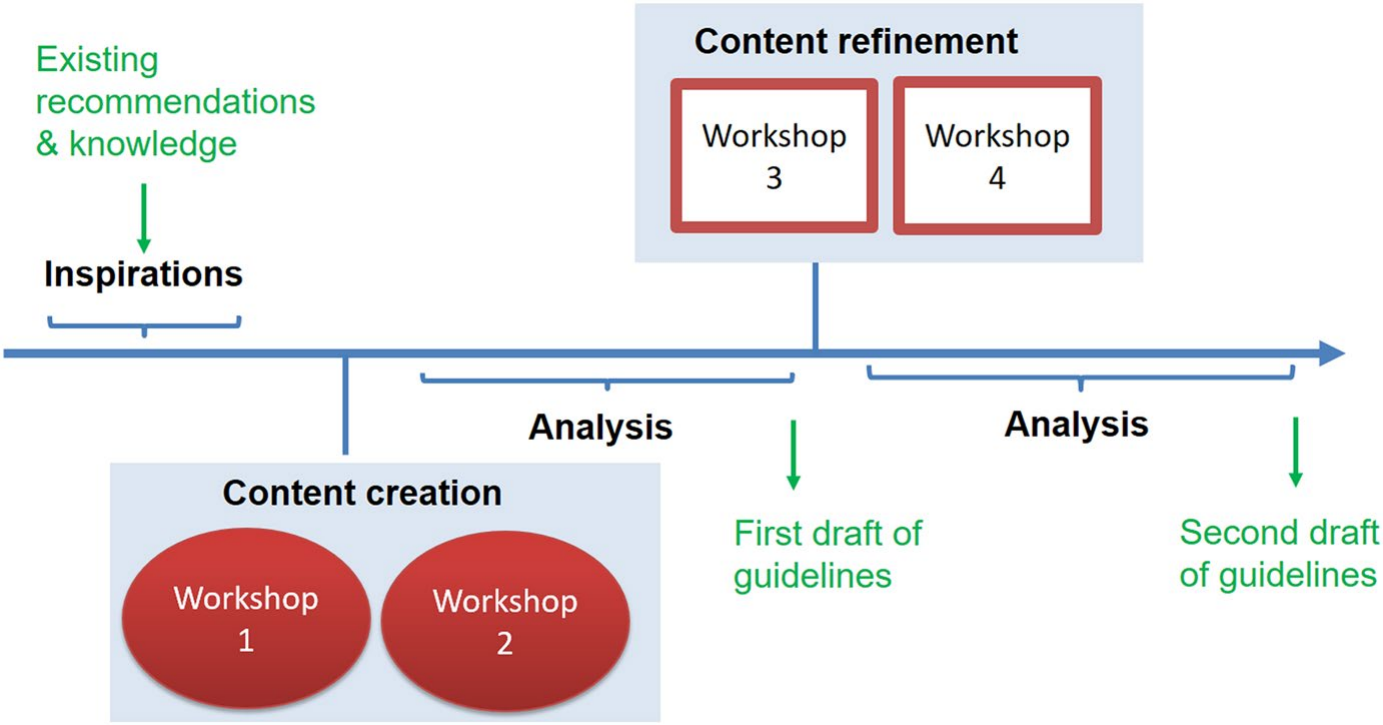
Guideline co-creation project process

The workshops were approved by the institutional review board of KU Leuven under dossier number G-2020011945. Prior to taking part in the workshops, participants received an information leaflet and signed an informed consent form. A detailed workshop protocol can be found on the Open Science Framework (https://osf.io/8upmb/). We conducted four co-creation workshops to develop the RI education guidelines: workshops one and two were dedicated to content creation and workshops three and four were dedicated to content refinement (Fig. 2, with more details available in Supplementary File I). We created ‘*inspirations’*—images or short pieces of text representing different recommendations—based on a compilation of existing ideas and recommendations about RI education from the preliminary steps (Lechner et al., 2020). The ‘inspirations’ served as ‘sensitization’ material; ‘sensitization’ primes participants to various ideas and promotes creativity in co-creation workshops (Sanders & Stappers, 2012; Sleeswijk Visser et al., 2005). The ‘inspirations’ sensitized participants to ideas about RI education elicited in the preliminary steps, and promoted different interpretations, as well as encouraging discussion of out-of-the-box, new ideas (Labib et al., 2021b). We sent the resulting ‘inspirations’ to all participants.

A week later, we conducted the first two workshops. During each workshop, we asked experts to create the content of RI education guidelines separately for (1) students, (2) researchers, and (3) other stakeholders, to ensure that relevant differences between target groups could be addressed. Furthermore, each workshop had a section dedicated to the role of providing directed advice and counseling for RI as a form of teaching about RI, to address forms of RI education that do not fall under the category of formal RI training. During these content creation workshops, we focused on generating a broad range of ideas for the content of the guidelines. Based on the discussions in the content creation workshops, we drafted a first version of the guidelines which we sent to participants of the content refinement workshops (i.e., workshops three and four). During the content refinement workshops, we asked participants to provide general comments, additions and concerns about the guidelines, for instance regarding redundancies, gaps, lack of clarity, conflicting statements. Following the content refinement workshops, we revised the guidelines further and sent them to participants to provide any additional comments or suggestions. Further details about the workshop proceedings, technical details, and facilitation can be found in Supplementary file I.

Each workshop lasted approximately 3 hours. Due to the COVID-19 pandemic, we conducted all workshops online using the virtual collaborative whiteboard software program MIRO (https://miro.com), and the video conference program Zoom (https://zoom.us/). The workshops were led by a facilitator (JT), as well as one or two co-facilitators (DP, BT, NS), and they were audio and video recorded, and transcribed. The role of the facilitator throughout the workshops was to guide the process of co-creation and ensure the inclusion of all participants’ ideas, without providing input to the content. The detailed program for each workshop can be found on the Open Science Framework (https://osf.io/9bztf/).

#### Analysis

KL, IL and NAB used inductive (Boyatzis, 1998) and deductive thematic analysis (Crabtree & Miller, 1992) to analyze the results of the ‘content generation’ and ‘content refinement workshops’, respectively, through an analysis-on-the-wall approach using MIRO as described by Sanders and Stappers (2012). The analysis was done collaboratively by the coders as described in Supplementary File I as is common for co-creation methods (Sanders & Stappers, 2012), so as to include various perspectives in the coding and enrich the interpretation and construction of codes and themes. Differences between coders therefore contributed to a more nuanced understanding of themes, and contributed towards an iterative analysis process. A detailed code book including the theme and subtheme labels, and detailed descriptions and illustrative quotes for each was collaboratively developed by KL and NE (https://osf.io/y3c5n/). The code book was created per topic discussed in each workshop, namely the RI education of students, researchers, other RI stakeholders, and RI counseling and advise (which came to encompass all informal RI education approaches). Using the insights from the analysis, we developed the first and second draft of the RI education guidelines. To assess thematic saturation, we compared the insights gained during the content creation workshops (i.e. the analysis results and resulting guidelines) with the recommendations compiled from the preliminary steps, based on the views of other stakeholders (i.e., Lechner et al., 2020). Any points from the previous steps which had not been discussed in the content creation workshops were added to the guidelines, marked in a different color (as can be seen in Supplementary File II), and fed into the content refinement workshops, so that the content refinement workshop participants could comment on them. A detailed description of these analysis steps can be found in Supplementary File I.

### Revision and Finalization of the Guidelines

After the co-creation workshops, the precise formulations of the recommendations in the guidelines needed further revision in order to be clear and usable (Labib et al., 2021b). We organized a revision working group, composed of TK, GW and KL, which prioritized, reorganized, and optimized the draft recommendations in the guidelines (please see https://osf.io/f9ghj/ for more details). The working group aimed to increase the understandability, implementability, and comprehensiveness of the guidelines. While revising the guidelines in the working group, we scrutinized the similarities and differences in the recommendations across the RI educational target groups, to ensure that the recommendations for each target group were relevant and appropriate.

We had intended to create four guidelines on topics that were determined together with stakeholders in one of the preliminary steps of the research (Labib et al., 2021c), namely the RI education of (1) students and researchers without a doctorate; (2) researchers with a doctorate; (3) research support staff and RI teachers; and (4) RI counseling and advice (as a form of education that falls outside of formal training). However, based on feedback from other research in the preliminary steps (Labib et al., 2021a) and the co-creation workshop participants about these categories, during the guideline revision process, we revised the categories to the RI education of (1) bachelor, master and PhD students, (2) post-doctorate and senior researchers, (3) other RI stakeholders, and (4) continuous RI education. This required some substantial changes to the 4th category; we reworded the title of the guideline on RI counseling and advice to explicitly include all forms of informal RI education and included insights from the workshops that were not only about RI counseling and advice, but also about other ways of raising awareness about RI within an institution. Additionally, we removed RI counseling and advice recommendations that were not related to RI education (e.g., those dealing with misconduct), based on participants’ suggestions.

Following this, we sent the guidelines to three external stakeholders with expertise in RI (MvdH, JPB and MM), for feedback on how to further refine the guidelines to improve their implementability. We instructed experts to provide us with concrete feedback that we used to revise the guidelines directly. To ensure that the feedback from participants of the co-creation project was well considered and understood in the revision process, we also sent the revised guidelines to the co-creation workshop participants as a final member check (Thomas, 2017). All but one of the participants responded with approval of the guidelines or some additional suggestions—which we then integrated into the guidelines. Suggestions from the experts and participants were mostly related to refining the formulations in the recommendations (e.g. to recommend the use of ‘diverse learning environments’, rather than ‘blended learning’ for RI training). Other suggestions were related to implementation, and were not aimed at revising the guidelines themselves. A full overview of the suggestions received can be found on OSF: https://osf.io/we6pq.

## Results

To adequately address the needs of students, researchers and research support staff, as well as incorporate various formal and informal approaches to RI education, we co-created four guidelines on RI education. Each guideline focused on a specific topic that we decided on together with various stakeholders (Labib et al., 2021a, 2021b, 2021c; Ščepanović et al., 2021; Sørensen et al., 2021), and then finalized in the further guideline development process. The guidelines focus on: (a) RI education of bachelor, master and PhD students (https://osf.io/z7m3v/); (b) RI education of post-doctorate and senior researchers, including all researchers with a doctorate ranging from early career researchers to full professors (https://osf.io/6d9ta/); (c) RI education of other institutional RI stakeholders (https://osf.io/ya3qj/); and (d) continuous RI education (https://osf.io/ambg3/). Guideline c focuses on stakeholders, other than researchers, who play an important role regarding RI. This includes people involved in developing and implementing RI policy at the institution, handling complaints, raising awareness about RI, or providing support or information to researchers for good research practice (e.g., research integrity officer, ombudsperson, data management officer). The exact tasks and responsibilities of RI stakeholders targeted in guideline c differ per institution and country in Europe, so the guidelines do not provide descriptions of each role in detail.

Guidelines a–c focus on the steps institutions can take to provide successful education to various target groups, while guideline d focuses on approaches for RI education other than formal RI training. To ensure that the guidelines are flexible enough to be incorporated in different institutional and country settings, we refrained from prescribing specific training aims or content in them, nor a specific theory about RI education. Instead, the guidelines provide higher level recommendations which need to be further operationalized and tailored to the local context of the institution. Institutions with many resources and already existing RI education policies in place may be able to include many of the recommendations directly, while those with fewer resources or existing RI infrastructure will need to phase the recommendations in slowly over time. We do not provide instructions for institutions on how to do this, because the order and manner of implementing recommendations will depend on the specific institutional context, but we provide ‘in practice examples’ for different recommendations which can serve as inspiration for institutions on where to get started. The main recommendations from each guideline are shown in Table 2, whereas the full versions are available on the Open Science Framework using the links described above and under https://osf.io/z7m3v/, https://osf.io/6d9ta/, https://osf.io/ya3qj/, https://osf.io/ambg3/ (where the ‘in practice examples’ can also be found). As can be seen, there were commonalities in the guidelines across target groups, but there were also some important points of distinction that merit discussion.

**Table 2:** Key recommendations from the guidelines on RI education

Guideline title	*a. Guidelines for research institutions on the RI education of bachelor, master, and PhD students*
Recommendations	1. Integrate mandatory RI education into the bachelor and master curriculum 2. Deliver a mandatory RI course at the start of the PhD trajectory 3. Provide PhD students with follow-up elective courses on RI 4. Organize opportunities to discuss RI informally 5. Provide train-the-trainer education and basic qualifications for RI trainers 6. Use diverse learning environments, combining online and in-person elements in RI education 7. Focus on students’ actual experiences with research rather than merely addressing theory in RI education 8. Motivate and reward students to actively take part in RI education 9. Evaluate educational programs

Guideline title	*b. Guidelines for research institutions on the RI education of post-doctorate and senior researchers*
Recommendations	1. Deliver mandatory training about RI for researchers starting new positions 2. Provide researchers with follow-up specialized training on RI 3. Involve senior researchers in the RI training of students and junior researchers 4. Organize opportunities to discuss RI informally 5. Provide train-the-trainer education and basic qualifications for RI trainers 6. Use diverse learning environments, combining online and in-person elements in RI education 7. Consult with researchers about their RI education needs and tailor education accordingly 8. Motivate and reward researchers to actively take part in RI education 9. Evaluate educational programs

Guideline title	*c. Guidelines for research institutions on the RI education of institutional RI stakeholders*
Recommendations	1. Provide institutional RI stakeholders who are not performing research with basic RI training 2. Organize events where RI stakeholders come together to ask questions, exchange experiences and discuss how to work together on RI 3. Provide train-the-trainer education and basic qualifications for RI trainers 4. Organize follow-up educational events when RI policies and regulations change 5. Provide opportunities for peer-to-peer learning about RI 6. Motivate and reward various RI stakeholders to actively take part in RI education 7. Evaluate educational programs

Guideline title	*d. Guidelines for research institutions on continuous RI education*
Recommendations	1. Provide researchers with educational RI resources to consult when needed 2. Show institutional commitment to provide continuous RI education 3. Provide researchers with contact persons who can support continuous RI education, by providing low-threshold, disciplinary-specific advice on day-to-day RI questions 4. Develop policies to foster responsible supervision and leadership 5. Develop policies for building a responsible research environment

### Commonalities Across Target Groups

#### Initial Mandatory RI Training

Across target groups, the co-creation participants recommended mandatory RI training to ensure that everyone in the institution is well-informed about RI. More specifically, co-creation participants thought that RI training should be mandatory when starting a new academic degree program (e.g. a bachelor, master, or PhD trajectory) or a new research position (e.g. new professorship, new postdoctoral research contract, etc.). Furthermore, participants stressed the importance of having RI trainers undergo train-the-trainer courses to ensure that they are not only aware of RI theory, but are also equipped with the necessary didactic skills and tools to train students and researchers. Participants also highlighted that other RI stakeholders such as ombudspersons and RI officers would benefit from educational activities about RI, although they did not explicitly mention formal training for this. In our revision working group, we proposed to extend the recommendation for formal basic training also to these RI stakeholders when starting new positions, to ensure that they have sufficient knowledge of RI to be able to support researchers with RI.

#### Follow-Up RI Training

The co-creation workshop participants recommended that all target groups should be provided with periodic follow-up RI training. At the bachelor and master level, they suggested that discussing RI in depth during the thesis research phase would be most appropriate. For PhD students, it was thought that follow-up courses on discipline-specific RI topics such as data management would be most helpful in supporting students’ research practice. Similarly, the co-creation workshop participants suggested that repeating follow-up disciplinary-specific training every 2–3 years to address specific RI issues such as new developments in research, would also be valuable for post-doctorate and senior researchers to keep up with the newest regulations and policies, as well as to refresh their knowledge and skills on RI. The same reasoning applied to other RI stakeholders such as RI officers, ombudspersons, and policy staff, to suggest that institutions should provide new training for these target groups every time new policies and regulations are introduced.

#### Informal Discussions About RI

The importance of informal RI discussions were highlighted during our co-creation workshops, where many participants thought that discussing RI experiences and problems in informal meetings together with colleagues, supervisors and supervisees, would be valuable for continuous RI peer-to-peer learning. However, some participants were concerned that it might be difficult for institutions to coordinate informal meetings. Therefore, in our revision working group, we recommended that institutions should stimulate and support departments and teams to organize informal events and integrate RI questions in them (e.g., by providing institutional awards for the best RI events), rather than coordinate this process themselves. The co-creation workshop participants agreed that RI education can contribute to a more responsible research culture, while an open research culture is a prerequisite for fruitful interactions during RI education. To take this consideration into account, all the RI education guidelines recommend that institutions develop policies that foster a responsible research environment (addressing community building, https://osf.io/7fn2x; diversity and inclusion, https://osf.io/fwa5c; managing competition and publication pressure, https://osf.io/ya3qj; and adequate education and skills training to researchers, https://osf.io/2p3vf), as discussed by the co-creation workshop participants and our revision working group.

#### Motivation, Incentives and Rewards

To motivate students and researchers to actively take part in RI education, our co-creation workshop participants suggested that RI educational events should emphasize the importance of RI (e.g., for research quality) and use a positive approach to RI (focused on promoting responsible research rather than discussing misconduct). This can involve highlighting the importance of RI education for researcher productivity, and professional and scientific success. A positive approach entails focusing on the real-life challenges faced in research practice rather than only teaching RI theory, telling trainees what to do, or focusing on the prevention of research misconduct. The co-creation workshop participants also stressed that institutions should provide suitable incentives and rewards to ensure students and researchers are actively engaged in RI education (e.g., free lunches, certificates, promotions). Our co-creation workshop participants additionally highlighted that it is not only researchers and students who should be rewarded for taking part in RI education and contributing towards improved RI, but also other RI stakeholders such as RI trainers and officers. Although motivation, incentives and rewards were recommended for all target groups, participants stressed that these should be tailored specifically for the target group as the same incentives and rewards might not work for everyone.

#### Evaluation of Educational Events

Evaluation was seen as crucial for the continuous improvement and update of RI education. Due to potential feasibility challenges in conducting objective outcome evaluations measuring changes in researcher behavior in every research institution, the workshop participants thought that process evaluations by research institutions would also be informative for evaluating educational events. They suggested using qualitative and quantitative measures for process evaluations. For instance, participants suggested to evaluate educational events using experiential data, such as how useful students perceive the event to be, as well as quantitative data not related to ‘effectiveness’, such as the number of individuals registering and attending optional RI trainings. Another quantitative measure that can be used, not mentioned by the workshop participants, is scores on assessments used to measure trainees’ knowledge, skills or attitudes related to RI. One of the experts we consulted with suggested that Kirkpatrick’s model of evaluations could be useful for evaluating RI education, since this widely used approach in the education domain looks at four dimensions when evaluating education including: (1) participants’ perceptions of the educational event, (2) skills and knowledge obtained, (3) any behavioural changes in participants following the education, and (4) the impact of the education on relevant institutional outcomes (Cahapay, 2021). Such an approach, therefore, helps to combine experiential and quantitative outcome measures.

### Points of Distinction Between Target Groups

#### Bachelor, Master, and PhD Students

Our co-creation participants thought that incentivizing RI education for bachelor, master and PhD students is relatively easy compared to other target groups. They suggested that providing students with tangible incentives—for example digital badges or other incentives tailored to students in different stages of their educational trajectory, disciplinary backgrounds and institutions—for completing trainings would suffice in motivating students to actively engage with RI education. Furthermore, they recommended providing all students with a substantial number of contact hours focused on RI (e.g., in the form of a complete course for PhD students), as this would ensure sufficient familiarization with RI at the start of their education about research, and would not be difficult to mandate.

#### Post-doctorate and Senior Researchers

Our co-creation workshop participants suggested that motivating post-doctorate and senior researchers to participate in RI education is difficult since researchers in these career stages are increasingly busy and have competing priorities. They stressed that institutional RI education policies should sufficiently address this concern to ensure engagement with RI education among researchers across seniority levels. Multiple recommendations on how to do this are offered in the guidelines. These include suggestions to consider RI and RI education in promotions and career assessments. The guidelines also offer other simpler suggestions such as labelling trainings as ‘Masterclass’ rather than a ‘training’ to make them sound more appealing to researchers. To reduce the burden that mandatory trainings would impose, many of the co-creation workshop participants suggested to provide this target group with small training events (e.g.. 1–2 h workshops), rather than full courses. It was also thought that a ‘bottom-up’ approach to RI training would be valuable to make RI training attractive and relevant for post-doctorate and senior researchers. Such an approach would involve consulting researchers beforehand to capture the RI topics they need support and help with (i.e. conducting a training needs analysis), and tailoring RI trainings accordingly. Tailoring trainings to trainees’ needs was thought to be especially relevant considering that very experienced senior researchers might have different training needs compared to researchers in earlier stages of their career.

#### Other Institutional RI Stakeholders

Co-creation workshop participants mentioned that peer-to-peer learning is likely most suitable for the RI education of other institutional RI stakeholders (e.g., ombudspersons, RI officers, trainers, policy makers, etc.). Participants suggested that research institutions foster peer-to-peer learning by supporting the organization of peer consultation meetings and other informal events where various RI stakeholders can come together to share experiences about RI and learn from each other. However, participants did recommend formal RI training for RI trainers, focusing not only on RI theory but also on didactical skills. They highlighted that national and European level support groups and networks for institutional RI stakeholders would be valuable to address the lack of availability of RI resources at some institutions, as well as fostering the sharing of experiences across institutions and countries. Many participants suggested that it would be helpful to share RI cases and educational materials in such a network in order to learn from each other and avoid ‘reinventing the wheel’. In our revision working group, we operationalized this recommendation to directly address research institutions, by suggesting that institutions provide RI stakeholders with opportunities to engage in peer-to-peer learning (e.g., by hosting networking events, providing funds or time to employees, etc.).

### Additional Measures to Increase Awareness About RI

Our co-creation workshop participants stressed that raising awareness about RI requires more than one-off RI trainings; therefore, in our revision working group, we decided to dedicate one of the RI education guidelines to continuous RI education to highlight this concern. Stimulated by advice from one of the experts we consulted in the guideline revision process, we decided to explicitly state that our definition of ‘RI education’ is broad and entails all means of creating awareness about RI—rather than only constituting formal education—in the preamble of the guidelines. The guideline on continuous RI education incorporates the co-creation workshop participants’ recommendations regarding institutional commitment to RI education, provision of necessary educational resources, creation of policies in the institution on building a responsible research environment, inclusion of responsible supervision and mentoring, and provision of low-threshold advice to researchers about RI through informal RI ‘champions’ or ‘stewards’ as a means of increasing awareness about RI. Regarding our recommendations in this guideline for building a responsible research environment and fostering responsible supervision, our continuous RI education guideline links to more detailed guidelines that we are developing in separate topics on the themes of research environment and supervision (Pizzolato et al., 2021, 2022).

## Discussion

We co-created institutional guidelines together with various research stakeholders regarding the RI education of (a) bachelor, master and PhD students, (b) post-doctorate and senior researchers, and (c) other institutional RI stakeholders, as well as (d) continuous RI education. These guidelines can help research institutions develop an overarching strategy for RI education that includes various educational approaches and addresses all relevant target groups. Across target groups, our guidelines indicate that institutions should organize continuous RI education using multiple formal and informal educational events (e.g., workshops, courses, informal discussions, etc.) and use target-group-appropriate incentives and rewards to actively motivate trainees to stay engaged in RI practices, for instance by including participation in RI education in promotion procedures for senior researchers. Furthermore, the guidelines suggest that education should focus on the concrete needs and practical challenges that participants deal with—a finding supported by a recent systematic review (Katsarov et al., 2022)—and use regular process evaluations to ensure constant updating and improvement. Moreover, our guidelines highlight that a holistic RI education approach will not only require provision of formal RI trainings, but also additional educational approaches (e.g., responsible supervision) so as to support continuous education.

Given that current RI education often consists of stand-alone courses on RI (Abdi et al., 2021a, 2021b), the implementation of continuous RI education will require substantial effort and commitment by research institutions to organize, design and deliver additional RI training events to various target groups. Although this could be perceived as a high burden by research institutions (Sørensen et al., 2021), we believe this commitment is necessary given that it is highly unlikely that a single course or workshop will be sufficient in influencing trainees’ perceptions and behaviors relating to RI (Kalichman, 2014). However, further empirical research on the effects that RI education has on researchers’ behavior is urgently needed to confirm this. To increase the feasibility of providing continuous RI education, institutions could make use of learning approaches utilizing different learning mediums where possible; they could consider using already existing openly accessible online RI trainings and resources, they could integrate relevant RI discussions in existing research courses, workshops, and department meetings, or they could cooperate with external trainers and institutions to share the provision of RI education. This recommendation is supported by evidence suggesting that blended learning approaches are highly effective for ethics instruction (Todd et al., 2017).

Almost every stakeholder we talked to, not only in the co-creation workshops, but also in the preliminary steps of the research process, agreed that some form of mandatory education on RI was needed across institutions. What some disagreed about was the form that this training should take (e.g., a full course versus a one hour workshop to update researchers about a new research development). While it can be assumed that mandating RI training for senior researchers is likely to meet resistance (Fanelli, 2019; Labib et al., 2021a), our co-creation workshop participants recommend mandatory RI training for senior as well as junior researchers. This is based on the view that mandatory training is the only way to ensure that all researchers—rather than only those who already consider RI as important—take part in RI education. Furthermore, training more senior researchers, especially regarding how to lead, manage and mentor their research teams responsibly and foster responsible research practices that warrant rigor, reproducibility and research quality, plays a crucial role in shaping institutional cultures including rigor, reproducibility, and integrity (Antes et al., 2016, 2019; McIntosh et al., 2020; Pizzolato et al., 2022). Our participants provided suggestions about how to provide training that is appropriate to different contexts (e.g., a session for seniors researchers to discuss the implications of new laws and policies, such as the General Data Protection Regulation, GDPR).

To reduce potential resistance and to ensure that trainees are actively engaged in RI education, the RI education guidelines stress the importance of providing suitable incentives and rewards for participating in RI training (e.g., tying RI education to promotions, using tangible rewards, etc.). Our guidelines further suggest to tailor incentives and rewards to their target group: a finding that is in line with existing literature suggesting that effective incentives and rewards are different for junior than for senior researchers (Fanelli, 2019; Labib et al., 2021a). Rewarding and incentivizing participation in RI education is also in line with other existing initiatives in the research community which state that researcher evaluations should consider a broader range of contributions and should value responsible research practices (Aubert Bonn & Bouter, 2021; Moher et al., 2020).

Motivation to actively participate in training will also depend on the extent to which the RI training appeals to the needs of each target group. Therefore, our guidelines stress the importance of providing RI education that focuses on the specific needs and challenges of the education target group. Focusing on real life cases of RI dilemmas that come up in research practice when teaching students about RI can help increase the relevance of RI training and has been suggested by others as well (Fanelli, 2019; Kalichman, 2014; Katsarov et al., 2022). Our recommendation to use a training-needs-analysis to ensure that post-doctoral and senior researchers can determine what should be included in their RI trainings and how, rather than following trainings focusing on methods and context predetermined as relevant for them by trainers, has to our knowledge, not been discussed in previous literature. However, we believe that such an approach is important, particularly to prevent researchers from perceiving RI training as a box-ticking exercise (Labib et al., 2022). Especially considering that researchers of various disciplines and ranks (e.g., full professors as compared to less experienced post-doctorate researchers) may have different needs (ENERI, 2017), using such a bottom-up, tailored approach to RI education is likely to be valuable, albeit the associated financial and time costs present challenges for implementation.

To ensure that RI education is continuously updated and improved over time, the RI education guidelines emphasize the importance of evaluating RI educational events. However, our results also suggest that evaluating educational events on their effects on researcher behavior will likely be difficult, if not impossible, indicating the need for institutions to engage in subjective process evaluations (e.g., on perceptions of training usefulness) over outcome-oriented evaluations (e.g., relating to changes in actual behaviors). This approach to evaluation might seem unsatisfactory for trainers who would like to develop RI trainings based on solid empirical outcome research, as well as for institutions who would like to know that their RI education policies are actually impacting research practice. However, we would argue that it is not the responsibility of single research institutions to provide full insight into what makes RI education effective; a focus on subjective process evaluations is more feasible and can still provide valuable information to trainers. For instance, evaluations on subjective data like stakeholder experiences can provide valuable information about the contextual mechanisms and processes that influence the success of educational initiatives (Hamza et al., 2020). Of course, if institutions have the possibility and means to also conduct behavioral outcome-oriented evaluations, that can be beneficial in ensuring that RI education improves trainee learning, skills development, and behavior change. Re﻿lying on process outcomes in evaluations for those institutions unable to conduct behavioral outcome-oriented evaluations is likely to be more acceptable when the behavioral effects of the educational approach have already been documented in the literature. There are substantial current efforts to find strategies to measure RI training effectiveness on outcomes such as improvements in moral reasoning and changes in behavior (e.g. Abdi et al., 2021a, 2021b; Katsarov et al., 2022; Watts et al., 2017); these can provide institutions with information on the effectiveness of RI education and supplement institutional efforts in process evaluations of various educational programs.

### Strengths and Limitations

The guidelines we discuss in this paper are—to our knowledge—the first to provide an overview of what to include in research institutions’ overarching RI education strategy. The guidelines are a result of an iterative co-creative research process, involving various potential lead-users from different parts of Europe. The co-creation workshop methods we have used have allowed us to incorporate a wide range of perspectives in the guideline, including heterogeneity in participants from high and low resource institutions and countries in Europe. Further research is needed to explore the relevance  of the guidelines in other settings such as  in low and middle income countries.

As such, the guidelines have been developed with a focus on incorporating various research stakeholders’ actual RI education needs and perspectives. The qualitative approach to the guideline development process allowed us to understand stakeholders’ perspectives about RI education in depth and in a nuanced way. The participants of the co-creation workshops represent the guideline lead-users (e.g., RI officers and trainers) rather than end-users (e.g., junior and senior researchers). We decided to focus on lead-users since the intensity of co-creation workshops limits the number of participants that could be included in them (Sanders & Stappers, 2012) and since the guidelines should be convincing for policymakers to make them useful for implementation. Despite the fact that we could not include all types of stakeholders as participants in the co-creation workshops, we consider that the guidelines still provide a comprehensive and diverse user input given the engagement of both lead-users and end-users in our preliminary work (Labib et al.,2021a, 2021c; Roje et al., 2021). Nonetheless, it might be that further engagement with end-users in the finalization of the guidelines could have provided further insights which would have been relevant for the guidelines, particularly regarding differences in the guideline content across education target groups. Furthermore, our approach might be limited by the fact that we did not provide an open call for feedback on the guidelines.

It would be valuable to obtain insights on a larger sample of experts’ thoughts on the importance, relevance and feasibility of the guidelines, using quantitative means. Actual testing of the guidelines in a number of research institutions will be necessary to further refine the guidelines and make them implementable on a large scale. Such testing can provide insights about how the recommendations can be implemented with few resources. A pilot study can also help to create a more comprehensive and robust set of ‘best practice’ examples for the recommendations in each guideline. Institutions interested in using the guidelines will need to take into account costs, local capacity, cultural issues, and context-specific factors during implementation of the guidelines (Horbach & Sørensen, n.d.; Konach et al., 2022). Implementation of the recommendations will likely vary between institutions which already provide some RI educational programs and those that do not.

## Conclusions

Our work provides experience-based co-created guidance to research institutions on important considerations for developing a successful RI education strategy. Our guidelines on RI education address the needs of students, researchers and other RI stakeholders, and take into account various approaches to RI education. In the guidelines, we recommend mandatory RI training; follow-up refresher training; informal discussions about RI; appropriate rewards and incentives for active participation in RI education; and evaluation of RI educational events across target groups. Each of our four guidelines can be considered a distinct tool that institutions can access, adapt and implement to meet their institution-specific RI education needs. Research institutions across Europe can use our guidelines as tools to strengthen their RI education efforts and consequently contribute towards better quality and more trustworthy research.

## Supplementary Information

Below is the link to the electronic supplementary material.

Supplementary file1 (DOCX 1090 KB)

Supplementary file2 (DOCX 22 KB)

Supplementary file3 (DOCX 57 KB)

Supplementary file4 (DOCX 57 KB)

Supplementary file5 (DOCX 56 KB)

Supplementary file6 (DOCX 49 KB)

Supplementary file7 (DOCX 48 KB)

## Data Availability

Due to privacy reasons, the co-creation workshop transcripts used for this article are not publicly available. However, we have made the anonymized code book, including quotes, publicly available on the Open Science Framework: https://osf.io/y3c5n/. We excluded all descriptions about the characteristics of the quote owners (e.g. country, role, gender), to prevent the identifiability of the data, considering that the majority of the co-creators are acknowledged in this paper.

## Abbreviations

RI: Research integrity
RRPs: Responsible research practices
QRPs: Questionable research practices
