# Supplementary material for: Co-creating Research Integrity Education Guidelines for Research Institutions
Source: Sci Eng Ethics. 2023 Jul 20;29(4):28. doi: 10.1007/s11948-023-00444-2 (PMC10359202; doi:10.1007/s11948-023-00444-2)
Supplement: Supplementary file 1 — Supplementary file1 (DOCX 1090 KB) [file 11948_2023_444_MOESM1_ESM.docx]

**Appendix I: Detailed methodology used for the SOPs4RI co-creation workshops**

An overview of the demographics of participants included in each co-creation workshop can be found in Table 1.

*Table 1: Characteristics of participants included in each co-creation workshop*

| **Number of participants** | **Countries** | **Stakeholder types** |
| --- | --- | --- |
|  |  |  |
| ***Workshop 1*** |  |  |
| 4 | Belgium, Sweden, Netherlands, Ireland | RI coordinator, Research manager |
|  |  |  |
| ***Workshop 2*** |  |  |
| 5 | Netherlands, Lithuania, Spain, Italy | Senior researcher, Research head, RI coordinator |
|  |  |  |
| ***Workshop 3*** |  |  |
| 4 | Sweden, Spain, Finland, Switzerland | RI coordinator, Research manager, Publisher |
|  |  |  |
| ***Workshop 4*** |  |  |
| 5 | Netherlands, Belgium, Ireland, UK, Germany | Senior researcher, Research manager |

The co-creation process used to develop the RI education recommendations consisted of a number of steps, including: creating *inspirations*, content creation, a first round of analysis, content refinement, and a second round of analysis (Figure 1). The role of the facilitator throughout the workshops was to guide the process of the co-creation and ensure the inclusion of all participants’ ideas, without providing input to the content. Some details on each step can be found below, while (1,2) provides the full overview of the methods used for the workshops.


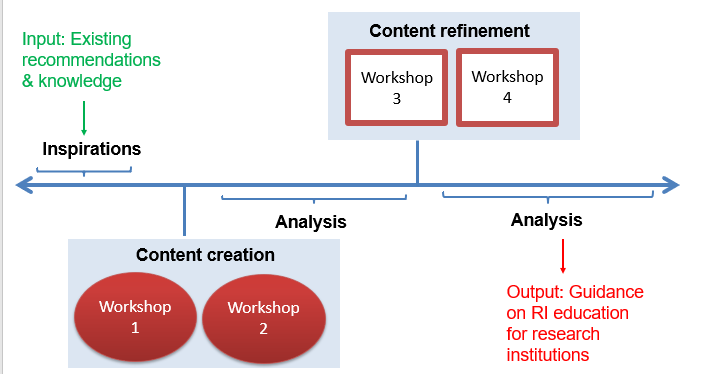


Figure 1 Guideline co-creation process in SOPs4RI project

*Inspirations*

To make use of existing recommendations about RI education compiled by the SOPs4RI consortium (3), we created ‘*inspirations’* – images and/or short pieces of text representing different recommendations – which we used to evoke ideas in the workshop participants without steering them into specific directions, as they could be interpreted in multiple ways (<https://osf.io/8dzxg/>). For instance, we used the text ‘knowledge’ and the image of a crystal ball to represent the possibility of focusing on the acquisition of knowledge, or reflective skills in education, respectively. To ensure participants’ familiarity with the *inspirations* during the workshops, the *inspirations* were sent to participants a week before the workshop. During this time, participants were asked to reflect on the *inspirations*, select three which they found most striking, and provide a rationale for their choices on the MIRO board (Figure 2).


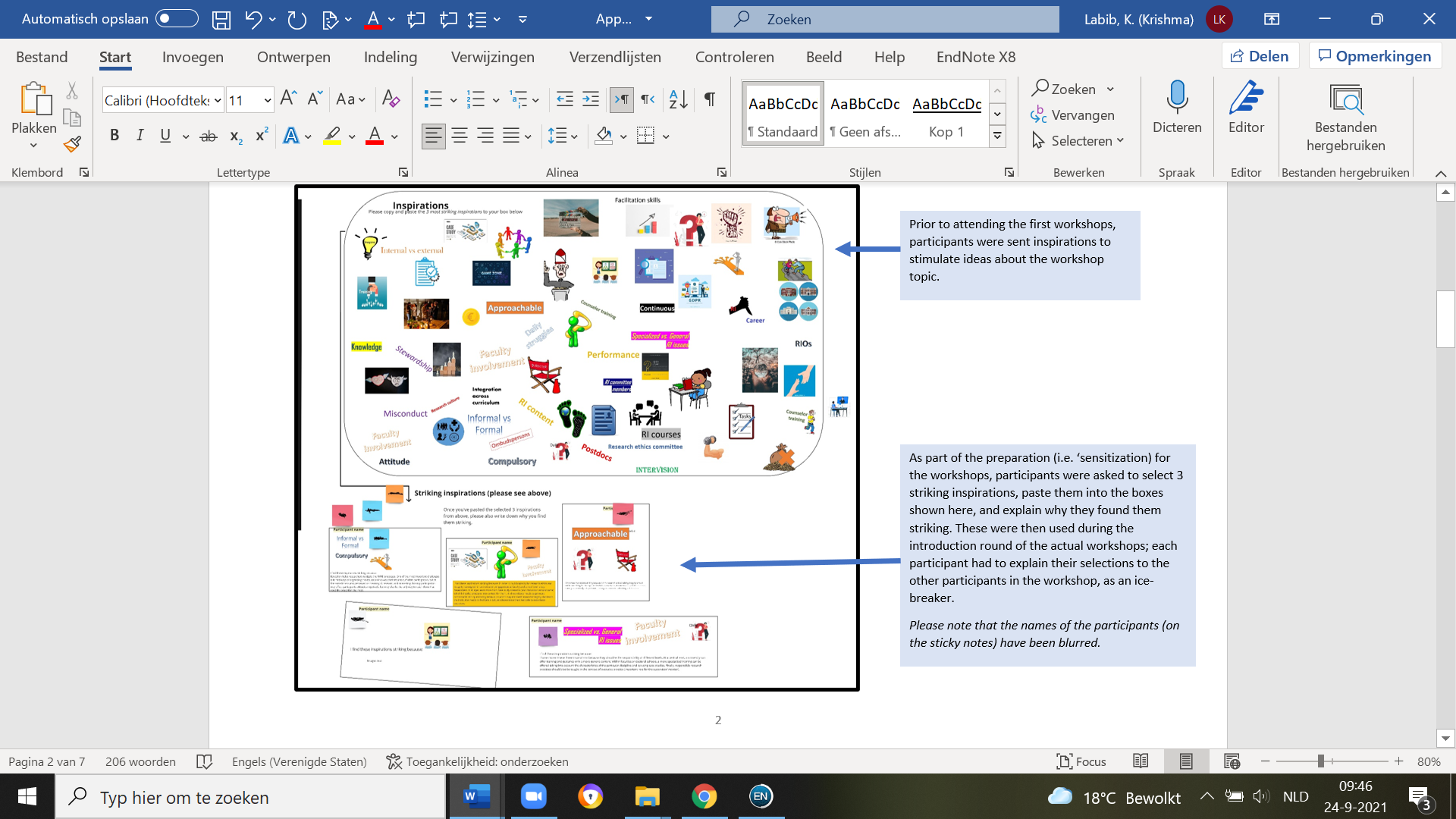


Figure 2 Use of inspirations in preparation of the co-creation workshops (i.e. the sensitization phase)

*Content creation*

During the ‘content creation’ workshops, we asked participants create the guideline content of four different topics related to RI education. We did this by asking participants to dream about what an ideal institutional education plan would be for: 1) students (including at the bachelor, master and PhD level), 2) researchers (post-doctorate to senior level), and 3) other research stakeholders (e.g. ombudspersons, RI officers and trainers); as well as to discuss how the institution should ideally provide 4) RI counseling and advice. Participants were encouraged to look at the *inspirations* for ideas (Figure 3). To optimize individual ideation (i.e. collect a large breadth of ideas from all participants), we first asked participants to individually think of as many ideas as possible and write them down on the exercise board. To foster an interchange of ideas and experiences, we then facilitated a collective discussion of their ideas. After these discussions, they could vote for the most important idea discussed, allowing for the revision of original ideas based on the interactions within the group. The group was asked to summarize the joint insights at the end of each exercise. Any differences of opinion were highlighted to be discussed in subsequent workshops. The full program for these workshops can be found here: <https://osf.io/9bztf/>. Following the workshop, we sent a summary of the workshop conclusions to the participants as a member check.


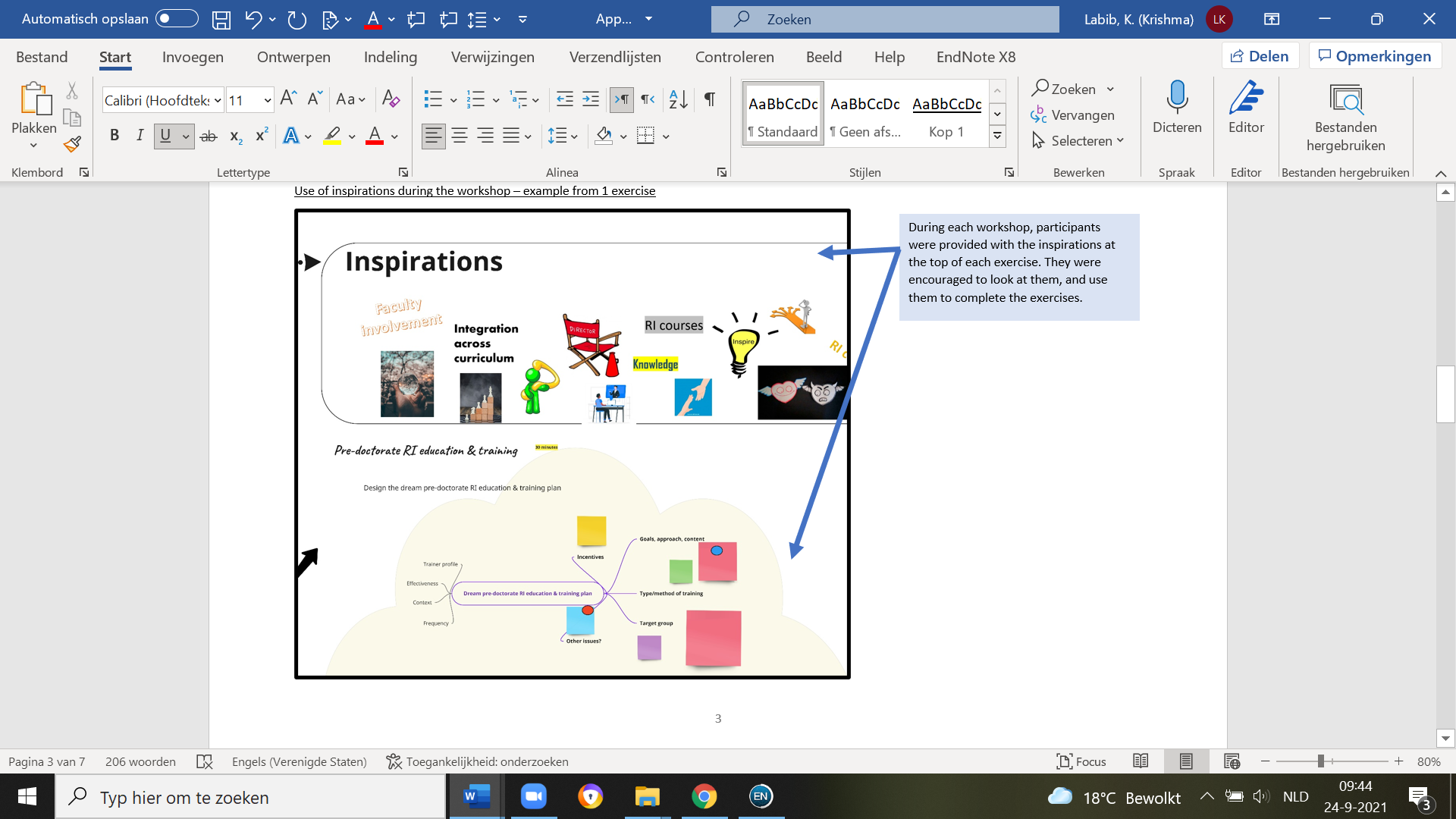


Figure 3 Example exercise used during the content creation workshops.

*First round of analysis*

We analyzed the data using inductive thematic analysis (4) through an analysis-on-the-wall approach as described by Sanders and Stappers (2012). We used transcripts of the workshops that we generated automatically via Amberscript (5). Two researchers – KL and IL – independently read through the workshop transcripts to identify quotes about the RI education recommendations per topic (i.e. 1) students, 2) researchers, 3) other RI stakeholders, and 4) RI counseling and advice), and pasted them on a MIRO board for analysis; KL also listened to the recordings of the workshop. The visual outputs of both workshops were also copied onto this MIRO board. The researchers met to conduct an *‘analysis workshop’*, during which they clustered all the data (i.e. quotes and visual outputs) per topic into themes. Each theme was subsequently assigned a label. The analysis results were visualized in an analysis poster per topic. Using the insights from the analysis, i.e. phrasing the themes in prescriptive terms and organizing them in a coherent order, KL developed a first draft of the RI education recommendations per target group, in collaboration with JT. When formulating the recommendations, we made them general enough that any context-related specificities were excluded. Discrepancies or differences in opinion among participants not related to context were highlighted in the guidelines for discussion in the follow up workshops. To ensure that this first draft adequately incorporated insights from the preliminary steps of the guideline development process (3), we added any additional recommendations from the preliminary work not yet discussed in the co-creation workshops to the guidelines, and marked these clearly as not originating from the workshops. The guidance documents were sent to participants of the ‘content refinement’ workshops a week in advance. In the preparation phase of the ‘content refinement’ workshops, participants were asked to reflect on how these guidelines would impact their institution.

*Content refinement*

The ‘content refinement’ workshops represented the ‘convergent’ phase of the co-creation process as described by Stelzle, Jannack, and Noenning (6). During these workshops, we focused on refining the RI education recommendations by asking participants to comment on the draft recommendations per topic (i.e. 1) students, 2) researchers, 3) other RI stakeholders, and 4) RI counseling and advice) (example shown in Figure 3). Participants were invited to provide general comments, additions and concerns about the recommendations (e.g. redundancies, gaps, unclarities, conflicting statements, etc.), rather than focus on specific terms and formulations. Additionally, they were invited to provide some best practice examples that could accompany the recommendations, as well as flag potential implementation challenges and opportunities. As in the content generation workshops, we first facilitated individual ideation, followed by a group interchange and building up of ideas, and the group summarized joint insights at the end of each exercise. We asked participants how to deal with any differences of opinion in the guidelines. A summary of the conclusions of the workshops were sent to participants following the workshop, as a member check.


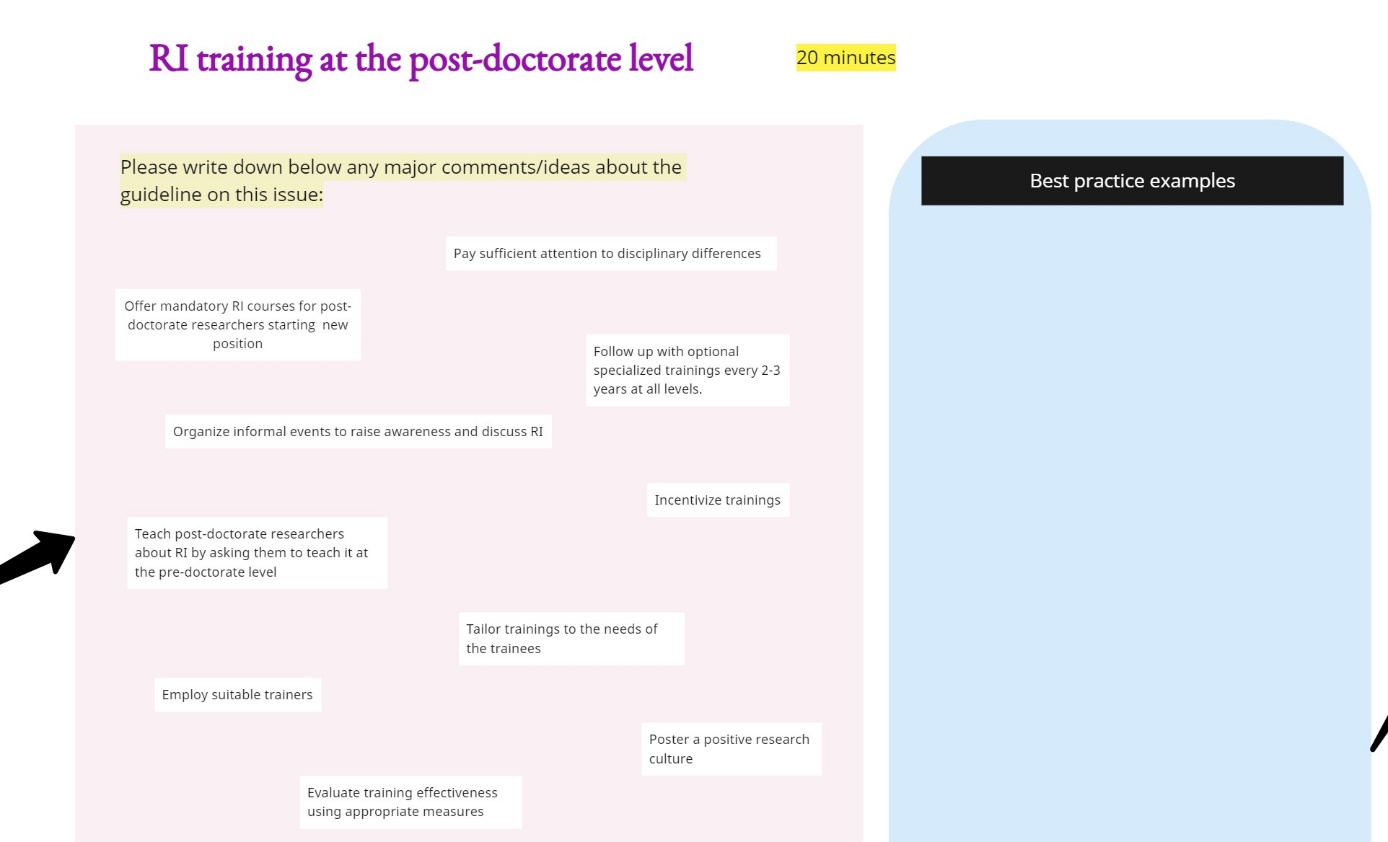


Figure 4 Example exercise used during the content refinement workshops

*Second round of analysis*

Deductive thematic analysis (7) was used for the analysis of the content refinement workshops, using an analysis-on-the-wall approach (8). After reading through the workshop transcripts (genderated automatically by 5) to identify quotes (with KL also listening to the audio recording of the workshops), and pasting these alongside the workshop visual outputs on a MIRO analysis board, KL, IL and NAB conducted another *‘analysis workshop’*. During this workshop, they deductively clustered the data into themes corresponding to first draft of the RI education recommendations per topic, as well as two additional themes on ‘implementation concerns’ and ‘best practice examples’. In some cases, the themes were slightly altered or expanded upon. Differences between the results from the content creation and content refinement workshops were visualized in analysis posters per topic. Based on the new insights from the content refinement workshops, KL revised the RI education recommendations. The recommendations were kept general enough that any context-related specificities were excluded. In case of any unresolved discrepancies or differences in opinion among participants, we tried to formulate the guidelines in a way that would allow the guideline users to interpret the guideline and tailor it in the way that they preferred. The revised recommendations were sent to all participants for another member check.

Additional analysis

To ensure the rigor of the analysis, RJ checked and made corrections in the automatically generated transcripts as we had deemed them to be ~90-95% correct in earlier steps. The earlier stages of the data analysis were revisited to scrutinize alignment between the overarching themes, subthemes and corresponding quotes (by KL and NE) as in Fereday and Muir-Cochrane (9). Based on this, a detailed code book including the theme and subtheme labels, and detailed descriptions and illustrative quotes for each, was collaboratively developed per target group by KL and NE (<https://osf.io/y3c5n/>).

**References**

1. Pizzolato D, Dierickx K, Tijdink J, Labib K, Lechner I, Bonn NA, et al. D4.4: Report on the Co-Creation Workshops [Internet]. 2021. Available from: https://sops4ri.eu/wp-content/uploads/D4.4_Report-on-the-Co-Creation-Workshops.pdf

2. Labib K, Pizzolato D, Stappers PJ, Evans N, Lechner I, Widdershoven G, et al. Using co-creation for guideline development - How why and when? OSF Prepr [Internet]. 2021; Available from: https://osf.io/cg3rw/

3. Lechner I, Tijdink J, Sørensen MP, Ravn T, Bendtsen A-K, Labib K, et al. D4.3: Second version of SOPs and guidelines [Internet]. 2020. Available from: https://sops4ri.eu/wp-content/uploads/D4.3_Second-version-of-SOPs-and-guidelines-1.pdf

4. Boyatzis RE. Transforming qualitative information: Thematic analysis and code development. Thousand Oaks: SAGE Publications Inc; 1998.

5. Amberscript. Transform your audio and video to text and subtitles [Internet]. 2021. Available from: https://www.amberscript.com/en/#

6. Stelzle B, Jannack A, Noennig JR. Co-design and co-decision: Decision making on collaborative design platforms. Procedia Comput Sci. 2017;112:2435–44.

7. Crabtree BF, Miller WF. A template approach to text analysis: developing and using codebooks. In: Crabtree BF, Miller WF, editors. Doing qualitative research. Newbury Park, CA: SAGE Publications Inc; 1992. p. 163–77.

8. Sanders E, Stappers PJ. Convivial toolbox: generative research for the front end of design. Amsterdam: BIS Publishers; 2012.

9. Fereday J, Muir-Cochrane E. Demonstrating rigor using thematic analysis: A hybrid approach of inductive and deductive coding and theme development. Int J Qual methods. 2006;5(1):80–92.
