# Supplementary material for: Co-creating Research Integrity Education Guidelines for Research Institutions
Source: Sci Eng Ethics. 2023 Jul 20;29(4):28. doi: 10.1007/s11948-023-00444-2 (PMC10359202; doi:10.1007/s11948-023-00444-2)
Supplement: Supplementary file 2 — Supplementary file2 (DOCX 22 KB) [file 11948_2023_444_MOESM2_ESM.docx]

**Appendix II: First version of RI education guidelines**

* Items in blue are based on other insights from the [anonymized] project, rather than the outputs of the [anonymized project name] co-creation workshops.

---------------------------------------------------------------------------------------------------------------------------

**Guidelines to research institutions on pre-doctorate research integrity education & training**

At the Bachelor/Master level:

1. **Provide training to students who do research**
2. **Integrate research integrity training into the curriculum (e.g. as part of thesis process), making it mandatory**

At the PhD level:

1. **Offer a mandatory course about the basics of research integrity at the start of the PhD**
   1. Employ trainers with general expertise in research integrity
   2. Empower trainees to speak up in their teams, by teaching them about institutional policies.
   3. Provide RI trainings as complete courses rather than one-off workshops
   4. Provide multidisciplinary trainings, but ensure that trainings sufficiently address the specific challenges faced in the disciplines of the trainees.
2. **Follow up with optional specialized courses throughout the PhD**
   1. Employ trainers with specialized expertise
3. **Supplement formal training with regular informal discussions at departments**
   1. Mix junior and senior researchers in some of these sessions.

At all pre-doctorate levels:

1. **Employ suitable trainers**
   1. Employ young and enthusiastic trainers whom the trainees can relate to
   2. Involve faculty in the delivery of trainings
2. **Ensure that training is continuous**
3. **Provide substantive contact hours for trainings**
4. **Emphasize practice over theory in trainings**
   1. Teach students the basic values of research integrity
   2. Focus on the daily practice of research, rather than emphasizing ethical theory
   3. Integrate relevant practical elements of research ethics issues into research integrity trainings
   4. Address cultural differences in the understanding of research integrity during training
   5. Discuss case studies and real-life examples during trainings
   6. Update research integrity courses based on trainees’ needs
5. **Use blended-learning formats, combining online and off-line training approaches**
6. **Provide tangible rewards, such as digital badges, to make trainees enthusiastic.**
7. **Ask students to reflect on research integrity in their theses, to evaluate the training effectiveness**
8. **Foster a positive research culture**
   1. As a prerequisite for training, to allow trainees to speak freely and engage in open discussions
   2. Through training.

**Guidelines to research institutions on post-doctorate research integrity education & training**

1. **Offer mandatory courses about research integrity basics for post-doctorate researchers starting a new position.**
   1. Employ trainers with general expertise in research integrity
   2. Supplement the mandatory trainings with follow-up peer support meetings.
2. **Follow up with optional specialized trainings every 2-3 years at all post-doctorate levels.**
   1. Provide easily accessible online modules with specialized content.
   2. Employ trainers with specialized expertise
3. **Organize informal events to raise awareness and discuss research integrity.**
4. **Teach post-doctorate researchers about research integrity by asking them to teach about the topic at the pre-doctorate level**
5. **Incentivize trainings:**
   1. Label trainings as 'Masterclass' rather than ‘training’ to make them sound more attractive.
   2. Do not label trainings with normative titles such as ‘research integrity’, but rather use more relatable and neutral terms
   3. Integrate research integrity trainings into existing courses
   4. Link research integrity and research integrity training to funding, promotions, ethics review, etc.
   5. Highlight the importance of research integrity training in preventing reputational damage.
6. **Employ suitable trainers**
   1. Involve senior peers in the training delivery
   2. Employ young trainers who can communicate clearly
7. **Pay sufficient attention to disciplinary differences in trainings:**
   1. Provide training programs where different disciplines can come together.
   2. Provide disciplinary specific training at the department level.
8. **Tailor the trainings to the needs of the trainees:**
   1. Use a bottom up approach to training, where training focuses on the needs and questions of the trainees.
   2. Address cultural differences in the understanding of RI in training.
   3. Tailor the training approach based on the exact target group, as senior post-doctorate researchers will need a different strategy than more junior ones.
   4. Give researchers the space to share stories and challenges.
   5. Focus not only on the role of the researcher, but also that of the reviewer
   6. Update research integrity courses based on trainees’ needs
9. **Evaluate training effectiveness using appropriate measures such as**
   1. Performance (such as decision making in ethics cases)
   2. Knowledge (such as knowledge of human subjects regulation)
   3. Climate (such as the extent to which individuals endorse ethical behaviors)
   4. Products (such as self-reflection exercises)
   5. Organizational outcomes (such as a drop in the incidence of ethical violations)
10. **Foster a positive research culture**
    1. As a prerequisite for training, to allow trainees to speak freely and engage in open discussions
    2. Through training

**Guidelines to research institutions on research integrity education & training for research integrity personnel and teachers**

1. **Provide trainings, where personnel from various departments at the institution are brought together to share roles, experiences, and discuss how to work together.**
   1. Include: research integrity committee members, data management personnel, legal staff, library staff, research integrity trainers, researchers, policy and management staff, confidential counselors, etc.
   2. Teach staff the relevant skills needed for their role.
      1. Research integrity officers/committee members should address skills relevant for responsibly investigating allegations of misconduct.
      2. Confidential advisors/counselors/ombudspeople should address facilitation, mediation and interpersonal skills.
   3. Discuss case studies, relevant for the institution, to learn from each other.
      1. Less experienced staff should be presented with possible cases they might face.
      2. More experienced staff can present their own cases and discuss how they have dealt with them.
   4. Help staff understand researchers better
   5. Face-to-face trainings are more suitable here, but online sessions can be used to supplement the face-to-face components.
2. **Provide train-the-trainer trainings to research integrity trainers**
   1. Ensure that trainees learn about the foundations of research integrity and ethical theory
   2. Teach training methods to trainees
3. **Provide multidisciplinary trainings where disciplinary considerations can be discussed**
4. **Provide trainings regularly, with new trainings offered at least when policies/regulations/infrastructures change.**
5. **Include researchers in the trainings**
6. **Facilitate the formation of European level support groups about research integrity to support peer-to-peer learning.**
   1. Facilitate the sharing of institutional resources with others.
7. **Hire enthusiastic trainers with research experience.**
8. **Commit strongly to research integrity training, also for staff**
   1. Evaluate the training programs to assess how helpful they are
9. **Reward RI teachers and support personnel for their work**
   1. Reward support staff with good career opportunities and appreciate their work.
   2. Reward researchers who also take on support roles.

**Guidelines to research institutions on research integrity counseling & advice**

1. **Appoint trustworthy trained official confidential counselors, familiar with research, whom researchers can turn to in case of doubts or questions per department.**
   1. Clearly communicate to researchers that counselling is confidential.
   2. In case of misconduct queries, the counselor should have some power and the tools to help the researcher that has approached them, in case that is desired by the researcher
   3. The official confidential counselors’ contact details should be published on the institutional website
   4. A clarification should be given on what researchers can and cannot expect from this contact person.
   5. The official confidential counselors could also be the first contact point for any researcher who is considering filing an allegation of misconduct.
2. **Research institutions should provide researchers with contact persons for advice on specialized/domain specific RI issues**
3. **Recruit volunteers to be research integrity stewards and to act as informal 'first aid responders' to researchers with research integrity questions, in order to guarantee that researchers have access to low-threshold counseling.**
   1. The RI stewards do not have to have undergone official counsellor training, but should be knowledgeable about and experienced with RI issues.
   2. The name and contact details of RI stewards should be made available to all staff at the faculty/department.
4. **Ensure that the counselors and research integrity stewards are visible, approachable and easy to find.**
5. **Provide an online help desk where researchers can pose simple questions about RI and obtain answers either directly and/or in the form of FAQs.**
6. **Have a strong institutional commitment towards providing RI support.**
   1. Allocate sufficient resources and time to counselors, both reactively and proactively.
7. **Include counselors & support staff in policy and education, so that counseling can improve policy and education and vice versa.**
8. **Offer people in support roles the possibility to climb the career ladder by offering higher positions.**
