# Supplementary material for: Co-creating Research Integrity Education Guidelines for Research Institutions
Source: Sci Eng Ethics. 2023 Jul 20;29(4):28. doi: 10.1007/s11948-023-00444-2 (PMC10359202; doi:10.1007/s11948-023-00444-2)
Supplement: Supplementary file 3 — Supplementary file3 (DOCX 57 KB) [file 11948_2023_444_MOESM3_ESM.docx]

**Appendix II: Second version of RI education guidelines**

## Sub topic/Guideline 1: Pre-doctorate research integrity training

**Title of skeleton guidelines:**

Guidelines on pre-doctorate research integrity training for research institutions

**Recommendations:**

**At the Bachelor/Master level:**

1. **Integrate research integrity training into the curriculum, making it mandatory**
2. As a part of the introduction to the curriculum
3. As a part of the thesis writing process
4. Providing adequate contact hours for students

*Explanation:* *Participants in both sets of workshops discussed the importance of starting research integrity (RI) training as early as possible. There was some disagreement in the first workshop about whether the starting point should be the bachelor or master level, since bachelor students might not have any actual experience with research, but some participant in the second workshop explained that all students including those at the bachelor level will experience research to an extent. In fact, there were even suggestions in the second workshop from one participant that starting at the bachelor level is already too late, since many students experience research for the first time in high school and might learn irresponsible research behavior already at that point. Points 1a and 1b were suggestions of where in the curriculum to place RI training; 1a was suggested in the second set of workshops, whereas 1b was mentioned in both sets. Furthermore, item 1c was added from the SoRs, but confirmed as important to ensure that students do not see RI training as something optional, but rather mandatory and important.*

**At the PhD level:**

1. **Deliver a mandatory course about the basics of research integrity at the start of the PhD**
2. Employ trainers with general expertise in research integrity or collaborate with trainers in other institutions
3. Empower trainees to speak up in their teams, by teaching them about institutional policies.
4. Provide RI trainings as complete courses rather than one-off workshops, providing adequate contact hours.
5. Provide RI training in multidisciplinary groups during which participants from different disciplines are given the opportunity to discuss and address the specific challenges faced in their disciplines.

*Explanation: Participants in both sets of workshops explained that in order to ensure that all PhD students who need RI training receive it – rather than only those who are interested in RI in the first place – it is important to deliver mandatory RI training. They suggested to do this at the start of the PhD to ensure that students had the basic awareness and skills about RI early on. Item 1a arose from discussions in the first workshop set, where some participants suggested that the most suitable person to deliver the mandatory training would be someone with general knowledge about RI, rather than someone with more specialized knowledge (e.g. about data management). In the second set of workshops, some participants were concerned that not all institutions might be able to employ their own trainers; therefore item 1a offers some flexibility and guides institutions with less resources to collaborate with other institutions or trainers. Item 1b was based on discussions in the first set of workshop, where some participants mentioned that awareness about policies and rules can empower students to speak up about RI to those higher in the hierarchy, and that this would be highly desirable. In the first set of workshop, it was already suggested that this basic RI training for PhD students should consist of a course, rather than a smaller event (item 1c). Finally, some participants in the first workshop highlighted the importance of providing the training in a multidisciplinary context, to allow exchange of experiences and cases from different disciplines in the training (item 1d).*

1. **Follow up with elective specialized courses throughout the PhD**
2. Employ trainers with specialized expertise or collaborate with trainers in other institutions
3. Refer students to existing educational resources such as codes of conduct, online training, or other relevant guidelines

*Explanation:* *It was already discussed by many participants in the first set of workshops that basic RI training at the PhD level needs to be supplemented with follow up courses on specific topics (e.g. data management) further on in the PhD. This is because as students progress in their research, they will uncover new RI questions and challenges. To allow students to follow the specialized courses that are most useful for them, these participants suggested to keep follow up courses optional. However, in the second set of workshops, one participant was concerned that it would be very difficult to coordinate the delivery and uptake of optional specialized courses. Item 2a was brought up in the first set of workshops, and slightly modified after the second set of workshops where some participants were concerned that not all institutions will have the means to hire their own trainers. Item 2b is based on suggestions from the second set of workshops, that referring students to resources can already be good enough when institutions do not necessarily have the means to offer follow up courses themselves.*

1. **Encourage and support informal discussions at departments or research teams to supplement formal training**
2. Mix junior and senior researchers in some of these sessions.
3. Foster multi-disciplinary discussions.

*Explanation:* *Item 3 was raised in the first set of workshops, where many participants highlighted the importance of sharing experiences and problems in informal meetings for RI education. They suggested to have mixed-rank groups for some of the sessions to allow for sharing of different types of experiences, and learning across ranks (item 3a). In the second set of workshops, some participants stressed that having multi-disciplinary informal discussions is especially useful as much of research today is multidisciplinary (item 3b).*

**At all pre-doctorate levels:**

1. **Employ respected, enthusiastic and qualified trainers**
2. Employ a set of trainers to ensure expertise in all aspects of RI are covered (e.g. ethics, data management, open science, etc.)
3. If possible, hire internal or external trainers
4. If not possible to hire  trainers, collaborate with trainers or training programs from other institutions
5. Involve faculty in the delivery of trainings

*Explanation:* *Participants in both sets of workshops discussed the importance of hiring suitable trainers for RI training. In the first set of workshop, participants mentioned that a suitable trainer has the following characteristics: young, good communicator, enthusiastic, researcher. In the second set of workshops, one participant emphasized the importance of ensuring that trainers are sufficiently trained and qualified to offer good RI education. Another participant in the second set of workshops explained that a good trainer of RI does not necessarily need to do research, but must learn enough about it to train others. Yet another participant explained that the most important feature of trainer is that they are respected by those they train, as if they are not, then the training material will not be taken up successfully. Taking these considerations together, we decided to not make any judgments in the guideline about the age or profile of the trainers, as different institutions can go for different options, but to emphasize the importance of hiring trainers that are sufficiently qualified, enthusiastic, and respected. Item 1a arose because some in the second set of workshops mentioned that a team of trainers with different types of expertise might be needed. Items 1b and 1c are based on insights from the second set of workshops about how internal trainers are most suitable since they know the local context best, but that it might not always be possible hire these. Item 1d is from results of the first set of workshops, where some participants suggested that it can be helpful to have senior colleagues deliver the trainings, to show support for RI and illustrate its importance for practice.*

1. **Use blended-learning formats to allow for continuous learning**
   - 1. Communicate to trainees that they are on a continuous path of research integrity training
     2. Ensure that trainees can turn back to the training material to look at the content later.

*Explanation:* *This item was discussed and agreed on in both sets of workshops. In the second set of workshops, some participants highlighted that blended learning formats are most suitable for continuous training, as they allow trainees to go back to training materials to look at the content again (item 2b), and since they allow for the formation of online support groups. Furthermore, in the second set of workshops, participants suggested to communicate to trainees what the added value of the blended-learning format is for continuous learning so they can make the best use of it (item 2a).*

1. **Emphasize practice over theory in RI education and trainings**
2. Consult with potential trainees on what to cover during training and update the training based on trainees’ needs
3. Teach students the basic values of research integrity
4. Focus on the daily practice of research, rather than emphasizing ethical theory
5. Integrate relevant practical elements of research ethics issues into research integrity trainings
6. Address cultural differences in the understanding of research integrity during training
7. Discuss case studies and real-life examples during training

*Explanation:* *One of the participants in the first set of workshops stressed that training should not focus on ethical theory, as that is not what is most interesting or relevant to students. Many other participants in both sets of workshop agreed with this, and suggested to emphasize the practical issues of RI in training, rather than focusing on ethical theory. Item 3a came up in the second set of workshops, where some participants highlighted that it might be useful to consult potential students about what to include in courses, to ensure that courses’ emphasis remain close to practice. Items 3b-3e were brought up in the first set of workshops, to explain that while some basic theory (e.g. about values) can be introduced to students, it should be integrated into the trainees questions about their own research. Item 3f was discussed in both sets of workshops, where there was strong agreement to use case studies and real-life examples to keep training programs close to practice.*

1. **Motivate trainees using tangible incentives and positive instruction**
2. Clearly communicate the purpose and value of research integrity training (e.g. improving research quality, helping with grants, etc.)
3. Explore what rewards and incentives motivate trainees and tailor these accordingly
4. Focus on a positive approach to research integrity rather than on research misconduct or on telling trainees what to do.

*Explanation:* *In the first set of workshops, some participants suggested to provide trainees with tangible incentives for training, such as digital badges, as that would be sufficient for this target group. However, in the second set of workshops, some participants explained that incentives and rewards should be tailored, as different trainees might appreciate different types of incentives. Therefore, they suggested to explore what rewards motivate participants and then tailor these accordingly (item 4b). Furthermore, items 4a and 4c were added based on discussions in the second set of workshops, where some participants highlighted that a good understanding of the benefits of RI training and a positive approach to RI can also motivate trainees.*

1. **Evaluate training programs**
2. Use subjective measures (e.g. trainees’ perception of course usefulness)
3. Use follow up measures (e.g. number of participants enrolled in elective courses)

*Explanation:* *In the first workshops, some participants mentioned that to evaluate training effectiveness, students could be asked to reflect on RI in their thesis. While there was no disagreement with this particular point in the second set of workshops, the participants there discussed how evaluating ‘effectiveness’ of courses through objective means is difficult and maybe not even possible. Therefore, they suggested to evaluate training based on subjective means (item 5a) or on simple objective measures not related to effectiveness (item 5b). Therefore, we reformulate this entire item in the guideline to remove the word ‘effectiveness’ and add some flexibility in how training programs can be evaluated.*

1. **Foster a positive research culture**
2. As a prerequisite for training, to allow trainees to speak freely and engage in open discussions
3. Through training.
4. Rather than telling researchers what to do during training, focus on giving awareness of RI standards and best practices, as well as enthusiasm and support to act with integrity

*Explanation:* *This item was discussed in the first set of workshops, but under the subtopic ‘post-doctorate RI training’. We added it to this subtopic as well, as it also applies here. Participants’ rationale for including this item was that “education and ‘good’ research culture have to be hand in hand”, since they influence each other. This was supported by the SoRs. Furthermore, item 6bi was added based on a comment in the second set of workshops that RI training should not be about telling researchers what to do but rather giving them the means and tools to act responsibly, to create a collaborative and healthy environment.*

| **Best practice examples:**   - Research integrity training program at University College London: <https://www.ucl.ac.uk/research/integrity/research-integrity-training-framework> - Committee on publication ethics resources: https://publicationethics.org/core-practices - ‘Science in action’ course at University Pompeu Fabra: <https://www.upf.edu/web/phd-biomedicine/science-in-action> - Editage educational resources: <https://www.editage.com/insights/> - Stockholm University’s ‘Research ethics for human sciences’ course: https://www.su.se/department-of-philosophy/education/courses-and-programmes/research-ethics-for-human-science-1.523153?eventopenforinternationalstudents=true&q=&xpanded= |
| --- |

*Additional remarks: The best practice examples above were mentioned by participants in the second set of workshops. There are likely many other best practice examples available, which can be added to the list here.*

*Participants also mentioned some implementation considerations for these guidelines in the second set of workshops including that:*

- *Targeting young researchers is helpful as they are the future of research and they will mentor future young researchers*
- *It would be optimal to start RI training already at the high school level, as students are first acquainted with research at that level*
- *Top down support for the guidelines is necessary for implementation*
- *Measuring training effectiveness is difficult.*
- *Supervisors and mentors play an important role in RI training*
- *The purpose of RI training has to be clear to everyone for implementation*
- *A balance is needed between supporting bottom up initiatives and providing top down support, but is difficult to achieve*
- *It might be difficult to account for disciplinary differences in general RI training courses*
- *Training should start with general issues and move to specifics later*
- *Follow up formal and informal training is difficult to coordinate and organize*
- *Standardized terminology should be used in the guidelines to ensure everyone understands all concepts*
- *These guidelines are already well developed.*

## Sub topic/Guideline 2: Research integrity training for post-doctorate and senior researchers

**Title of skeleton guidelines:**

Guidelines on post-doctorate research integrity training for research institutions

**Guidelines**:

1. **Deliver mandatory training about research integrity basics for researchers with a doctorate starting a new position.**
2. As part of the introduction package for new employees
3. Include  employees starting a new position at the same institution in the training
4. Recap the basics of research integrity in this training
5. If the researchers have not yet obtained research integrity training at the PhD level, ask them to follow a PhD research integrity course as well.
6. Employ trainers with general expertise in research integrity or collaborate with trainers in other institutions
7. Supplement the mandatory trainings with follow-up peer support meetings.

*Explanation: Participants in both sets of workshops explained that in order to ensure that all post-doctorate researchers receive RI training – rather than only those who are interested in RI in the first place – it is important to deliver mandatory RI training. For feasibility purposes, they suggested to mandate the training to incoming researchers and those who start new positions (i.e. are promoted) at the institution (items 1a and 1b). Items 1c and 1d were brought up in the second set of workshops, as a means to ensure that all post-doctorate researchers have sufficient background in the basics of RI. Items 1e was based on participants’ suggestion in the firs set of workshops that the most suitable person to deliver the mandatory training would be someone with general knowledge about RI, rather than someone with more specialized knowledge (e.g. about data management). In the second set of workshops, some participants were concerned that not all institutions might be able to employ their own trainers; therefore item 1e offers some flexibility and guides institutions with less resources to collaborate with other institutions or trainers. The last item (item 1f) was only discussed in the first set of workshops; participants highlighted that peer support meetings could be very helpful and feasible ways to ensure continuous RI learning.*

1. **Follow up with mandatory specialized trainings every 2-3 years at all post-doctorate levels.**
2. Use small events, like half-day workshops, rather than full courses.
3. Provide or refer trainees to easily accessible online modules with specialized content.
4. Employ trainers with specialized expertise or collaborate with trainers in other institutions.

*Explanation:* *In the first set of workshops, some participants suggested that in order to keep up with the newest regulations and policies and refresh researchers’ knowledge and skills about RI, it would be helpful to offer optional follow up training events, focusing on specific RI issues (e.g. data management). However, in the second set of workshops, many of the participants preferred to make the follow-up training events obligatory, to ensure that all researchers are up-to-date on RI. These participants thought that a 2-3 year interval between trainings would ensure that these follow-up events are not burdensome. Furthermore, they suggested to keep the training events small, also to reduce the burden (item 2a). Item 2b was discussed in both sets of workshops. Item 2c was brought up in the first set of workshops, and slightly modified after the second set of workshops where some participants were concerned that not all institutions will have the means to hire their own trainers.*

**3.  Use blended-learning formats**

1. Ensure that trainees can turn back to training to look at the content later
2. Discuss case studies, but with a focus on positive aspects of research integrity rather than research misconduct.

*Explanation: Item 3 was only discussed in the second set of workshops for this guideline, and therefore recently added.* *Due to participants’ suggestions that many of the items in the pre-doctorate training guideline also apply to this guideline, we added item 3a here to ensure some consistency. Item 3b was discussed in the second set of workshops.*

**4. Encourage and support the organization of informal discussions at departments or research teams to supplement formal training**

1. Mix junior and senior researchers in some of these sessions**.**
2. Foster multidisciplinary discussions

*Explanation:* *This item was raised in the first set of workshops, where many participants highlighted the importance of sharing experiences and problems in informal meetings for RI education. It was slightly altered in phrasing from ‘Organize informal events’ to ‘Encourage and support the organization of informal discussions’ to take into account some concerns raised by a few participants in the second set of workshops, that organizing informal discussions can be difficult to coordinate and arrange. Item 4a was taken from the guidelines on pre-doctorate training, as it also seems to apply here. Item 4b was based on discussions in both sets of workshops as many participants stressed that having multi-disciplinary RI discussions is especially useful as much of research today is multidisciplinary. After the first set of workshops, item 4b was a main item but we eventually decided to place it as a sub-item under item 4 based on the suggestion of one of the participants of the second set of workshops that this should not be a main heading, and the suggestion of another participant that multidisciplinary concerns are especially interesting to discuss in informal discussions.*

**5. Teach post-doctorate and senior researchers about research integrity by stimulating them to teach about the topic at the pre-doctorate level**

*Explanation:* *Item 5 was included based on the results of the first set of workshops, where it was discussed that when post-doctorate researchers have to deliver RI training, it is a means for them to progress in their own RI education as well. However, in both sets of workshops, and especially so in the second set, there was hesitancy about the usefulness of this guideline as many participants were afraid that if post-doctorate researchers are not sufficiently trained in RI and enthusiastic about it to begin with, it would be risky to ask them to train more impressionable junior researchers.*

**6. Motivate trainees to actively participate in training**

1. Convey clearly that research integrity is important for research quality and relevant for all researchers.
2. Label trainings as 'Masterclass' rather than ‘training’ to make them more attractive.
3. Integrate research integrity trainings into existing courses
4. Link research integrity and research integrity training to funding, promotions, ethics review, etc.
5. Highlight the importance of research integrity training in preventing reputational damage.
6. In case of resistance to training, consider not labelling trainings with normative titles such as ‘research integrity’, but rather use more relatable and neutral terms such as ‘research practices’

*Explanation: This point was highlighted in the SoRs and extensively discussed in both sets of workshops.* *It was repeatedly emphasized by many participants motivating trainees is especially difficult at the post-doctorate level, so this needs a lot of attention in the guideline. Initially the item was named ‘Incentivize training’, but a participant in the second set of workshops suggested that ‘incentive’ is not appropriate to use when we discuss mandatory training, suggesting that ‘motivating trainees’ is more appropriate. Item 6a was raised in the second set of workshops, while items 6b, 6e-6f were raised in the first set of workshops, and items 6c-d originate from the SoRs.*

1. **Employ respected, enthusiastic and qualified trainers**
2. Employ a set of trainers to ensure expertise in all aspects of RI are covered (e.g. ethics, data management, open science, etc.)
3. If possible, hire internal or external trainers
4. If not possible to hire  trainers, collaborate with trainers or training programs from other institutions
5. Involve senior peers in the training delivery

*Explanation:* *Just like for the guidelines on pre-doctorate training, participants in both sets of workshops discussed the importance of hiring suitable trainers for RI training of post-doctorate trainees. As such, this item mirrors the item on suitable trainers for the pre-doctorate training guideline. Item 1a arose because some in the second set of workshops mentioned that a team of trainers with different types of expertise might be needed. Items 1b and 1c are based on insights from the second set of workshops about how internal trainers are most suitable since they know the local context best, but that it might not always be possible hire these. Item 1d is from results of the first set of workshops, where some participants suggested that it can be helpful to have senior colleagues deliver the trainings, to show support for RI and illustrate its importance for practice.*

1. **Tailor the trainings to the needs of the trainees:**
2. Conduct a training needs analysis (TNA) to learn about your target groups’ needs and tailor training accordingly
3. Senior post-doctorate researchers might need a different training strategy than more junior ones.
4. Plan meetings with researchers, to discuss what should be covered during training and tailor training accordingly
5. Address cultural differences in the understanding of RI in training.
6. Give researchers the space to share stories and challenges.
7. Address all roles of good researchers in training including mentorship, reviewing, leadership, etc.
8. Have follow up meetings with researchers to discuss how to integrate research integrity considerations into their research
9. Ensure that training has an added value to trainees and communicate this value clearly (e.g. helping with grant application success)

*Explanation:* *The need to use a bottom up approach for training was highlighted in both sets of workshops for the guidelines at the post-doctorate level. Item 8ai was already brought up in the first set of workshops, but the idea to do a trainings needs analysis and hold meetings with trainees to discuss what to include in training (items 8a-8b) was brought up by some participants in the second set of workshops. Similarly, items 8f-8g were also brought up in the second set of workshops. Items 8c-8d were based on insights from the first set of workshops, while 8e was integrated from the SoRs.*

1. **Evaluate training programs**
2. Use subjective measures (e.g. trainees’ perception of training usefulness).
3. Use follow up measures (e.g. number of participants enrolled in optional training)

*Explanation: For this guideline, evaluation was not discussed in the first set of workshops. Instead, we integrated this item from the SoRs phrased initially as ‘Evaluate training effectiveness using appropriate measures’. However, the participants in the second set of workshops discussed how evaluating ‘effectiveness’ of courses through objective means is difficult and maybe not even possible. Therefore, they suggested to evaluate training based on subjective means (item 9a) or on simple objective measures not related to effectiveness (item 9b). Therefore, we reformulate this entire item in the guideline to remove the word ‘effectiveness’ and add some flexibility in how training programs can be evaluated.*

1. **Foster a positive research culture**
2. As a prerequisite for training, to allow trainees to speak freely and engage in open discussions
3. Through training
4. Rather than telling researchers what to do during training, focus on giving awareness of RI standards and best practices, as well as enthusiasm and support to act with integrity

*Explanation: Participants’ rationale for including this item was that “education and ‘good’ research culture have to be hand in hand”, since they influence each other.* *This was supported by the SoRs. Furthermore, item 10bi was added based on a comment in the second set of workshops, that RI training should not be about telling researchers what to do but rather giving them the means and tools to act responsibly, to create a collaborate and healthy environment.*

| **Best practice examples:**   - Data management seminars for senior researchers - Small research integrity workshops - Marie Curie research integrity programs for postdoctoral researchers: https://www.mariecuriealumni.eu/topics/research-integrity - Ghost, as a way to evaluate courses: https://ghost.org/ |
| --- |

*Additional remarks: The best practice examples above were mentioned by participants in the second set of workshops. It is unclear whether there are other best practice examples available; it would be helpful to look for these further.*

*Participants also mentioned some implementation considerations for these guidelines in the second set of workshops including that:*

- *Mandatory training can lead to a box-ticking mentality*
- *It is more difficult to make courses mandatory at the post-doctorate level compared to the pre-doctorate level*
- *Funders can help to incentivize RI training by requiring it*
- *It is difficult to focus on concrete research practice, rather than ethical theory, in general RI training since each discipline has different practices*
- *Evaluating training effectiveness through objective measures is difficult.*
- *Standardizing RI training at the post-doctorate level across Europe is difficult when there are no/few formal courses available*
- *Many of the items mentioned in the pre-doctorate RI training guidelines have been added here as they also apply for this target group.*

## Sub topic/Guideline 3: Training of research support staff & research integrity trainers

**Title of skeleton guidelines:**

Guidelines on training of research support staff & research integrity trainers

**Recommendations:**

1. **Organize formal and/or informal events where personnel from various departments are brought together to share roles, experiences, and discuss how to work together on research integrity.**
2. Include: research integrity committee members, data management personnel, legal staff, library staff, research integrity trainers, researchers, policy and management staff, confidential counselors, etc.
3. Ensure that staff are equipped with the relevant skills needed for their role.
4. Research integrity officers/committee members should address skills relevant for responsibly investigating allegations of misconduct.
5. Confidential advisors/counselors/ombudspeople should address facilitation, mediation and interpersonal skills.
6. Discuss case studies, relevant for the institution, to learn from each other.
7. Less experienced staff should be presented with possible cases they might face.
8. More experienced staff can present their own cases and discuss how they have dealt with them.
9. Help staff understand researchers better
10. Face-to-face trainings are more suitable here, but online sessions can be used to supplement the face-to-face components.

*Explanation:* *This item was discussed in the first set of workshops and was initially phrased as: ‘Provide trainings, where personnel from various departments at the institution are brought together to share roles, experiences, and discuss how to work together’. The rationale behind the item was that bringing various support staff together to discuss questions, cases and experiences would be very informative and help staff to work better together. In the second set of workshops, a participant explained that the term ‘training’ might not be appropriate here, considering that a more informal event might be more suitable for this type of peer exchange of knowledge and experience rather than ‘training’ which involves a more top down approach to education. The participant even mentioned that it is not suitable to discuss hiring official trainers for this target group – an additional item that we had put in the earlier version of this guideline, which we then deleted. To further account for this view, we reformulated item 1 to exclude the word ‘training’ and explicitly mention that the exchange can occur in a formal or informal event, leaving room for flexibility in implementation. In line with this, we also reformulated item 1b which was initially phrased as ‘Teach staff the relevant skills’, to ensure ‘Ensure that staff address the relevant skills’. In the earlier draft of this guideline (after the first set of workshops), item 1a was also partially mentioned as a separate item as ‘Include researchers in the training’, but we removed that item due to redundancy. The rest of the points under this item arose either directly from the first set of workshops (items in black) or the SoRs (items in blue).*

1. **Ensure that research integrity trainers are provided with train-the-trainer training by referring them to existing training programs or developing an in-house training.**
2. Ensure that trainees learn about the foundations of research integrity and ethical theory
3. Ensure that trainees are taught about training methods.

*Explanation:* *In the first set of workshops, some participants stressed that specific training is needed for trainers of RI, where both RI basics (item 2a) and training methods (item 2b) are taught. In the second set of workshops, there was agreement about the importance of the item, but some participants expressed concern that not all institutions will be able to provide their own train-the-trainer RI training. To account for this, after the second set of workshops, we formulated item 2 as ‘Ensure that RI trainers are provided with…’ (rather than the previous formulation of ‘Provide RI trainers with…), and item 2b as ‘ensure that trainees are taught’ (rather than the previous formulation of ‘Teach trainees about training methods’).*

1. **Provide multidisciplinary trainings where disciplinary considerations can be discussed**

*Explanation:* *Participants in both sets of workshops agreed on the inclusion of item 3, as they mentioned that many types of research considerations are relevant for multiple disciplines (e.g. ethical considerations related to research with humans are the same across many disciplines).*

1. **Organize training events regularly, with new trainings offered at least when policies/regulations/infrastructures change.**
2. Use examples and cases to illustrate new policies, regulations, and/or infrastructures

*Explanation:* *This item was discussed in both sets of workshop as important to include to ensure that staff are aware of the most updated policies/regulations and infrastructures. Item 4a was added based on some participants’ suggestions in the second set of workshops that policies and regulations are often boring, and need to be ‘brought to life’ using interesting cases and examples.*

1. **Facilitate the formation of and participation in European level support groups about research integrity to support peer-to-peer learning.**
2. Facilitate participation in online seminars and workshops
3. Facilitate the sharing of institutional resources with others.

*Explanation:* *The usefulness and importance of European level support groups for RI staff was highlighted by participants in both sets of workshops, as was the sharing of institutional resources with others (item 5b). Item 5a was added due to some suggestions in the second set of workshops that online events are especially helpful to deal with problems with mobility across countries.*

1. **Commit strongly to research integrity training, also for staff**
2. Include research integrity/ethics as a central value of the institution
3. Highlight the intrinsic (e.g. improved research quality) and extrinsic (e.g. in relation to grants) importance of research integrity for research

*Explanation:* *Item 6 is more of an implementation issue for the guideline, rather than a point directly related to the training of RI staff. However, it was mentioned as a point to include in the guideline in both the first and second set of workshops, since many participants exclaimed that without top down support, the guideline would not work. Items 6a and 6b were additions made in the second set of workshops, to help make the overall item more concrete.*

1. **Evaluate training programs**
2. Use subjective measures (e.g. trainees’ perception of event usefulness)
3. Use follow up measures (e.g. number of participants in an event)

*Explanation:* *For this guideline, the item on evaluation – item 7 – was brought up by two participants in the second set of workshops, who stressed that evaluation of training programs for RI personnel & teachers was just as valuable as for other target groups. Therefore, we added this item to this guideline and formulated it in the same way as for the guidelines on RI training for pre-doctorate and post-doctorate researchers.*

1. **Reward RI teachers and support personnel for their work**
2. Reward the involvement of support staff, recognise their involvement in teaching RI in their career assessments, and appreciate their work.
3. Reward researchers who also take on RI support roles (e.g., confidential advisors, ombudsperson, etc.).

*Explanation:* *This item and its subpoints were brought up in the first set of workshops, and there was agreement on its importance in the second set of workshops. Although the item is not directly about training, it is important issue that is likely to have a significant influence on the implementation of this guideline.*

| **Best practice examples:**   - ERION: <https://www.earma.org/about/governance/thematic-groups/ethics-and-research-integrity-officer-network-erion/> - EU project Recaphe: <https://recaphe.eu/> - EURASHE: <https://www.eurashe.eu/> - EURAXESS: https://euraxess.ec.europa.eu/ |
| --- |

*Additional remarks: The best practice examples above were mentioned by participants in the second set of workshops. There are likely many other best practice examples available, such as materials from the VIRT2UE project, which can be added here.*

*Participants also mentioned some implementation considerations for these guidelines in the second set of workshops including that:*

- *It is less suitable to use the word ‘training’ for this target group, as exchange of knowledge is more suitable here rather than top down training*
- *Top down support is crucial for the implementation of this guideline*
- *Making RI a central strategy of the institution will ensure that sufficient time, resources and personnel are allocated to its implementation.*
- *Evaluating training programs is difficult*
- *COPE can help SOPs4RI with organizing European-level webinars*
- *Piloting the guidelines would be very helpful*
- *These guidelines are already well-developed.*

## Sub topic/Guideline 4: RI counseling and advice

**Title of skeleton guidelines:**

Guidelines on research integrity counseling & advice for research institutions

**Recommendations:**

1. **Appoint trustworthy trained official confidential counselors, familiar with research, whom researchers can turn to in case of doubts or questions per department or research teams.**
2. Ensure that counselors are knowledgeable about all relevant policies and guidelines at the international and local level
3. Have higher management endorse the trustworthiness of the counselor.
4. A clarification should be given on what researchers can and cannot expect from this contact person.
5. Set up a procedure for handling conflicts of interest relating to the role of the confidential counselor.

*Explanation*: *This item was raised in the first set of workshops, and expanded on in the second set of workshops. Items 1a-1b, and 1e were discussed in the second set of workshops. Items 1c and 1d are integrated from the SoRs. Initially, we had also put another item under here, as an outcome of the first set of workshops, stating that institutions should ‘clearly communicate to researchers that counseling is confidential’.* *However, a participant in the second set of workshops mentioned that in some countries, confidentiality cannot always be guaranteed as counselors might have a legal obligation to report misconduct cases. Therefore, we removed that item, and hope to have further addressed this concern under item 1d (from the SoRs).*

1. **Research institutions should provide researchers with contact persons for advice on specialized/domain specific RI issues (e.g. privacy officers, librarians, etc.)**

*Explanation: This item was not discussed in the first set of workshops, but was integrated into the guidelines from the SoRs (based on the results of the focus groups). In the second set of workshops, participants mentioned that they did not see the difference between this item and the previous one on confidential counselors. However, we decided to keep this item because it is rather different from item 1, which is about general RI counseling, as it is focused on specialized RI issues which general RI counselors might not have sufficient expertise in. To highlight this, we now provide some examples of specialized RI contact persons in the item, i.e. privacy officers, librarians, etc.*

1. **Ensure that needed advice is provided in a timely manner and with sufficient follow-up.**

*Explanation**: Some participants in the second set of workshops mentioned that the guideline was missing some information about what the institution should require about the quality of the counseling provided. Item 3 was added to address this point. Some participants explicitly stated that good counseling is timely and provides sufficient follow up.*

1. **Recruit volunteers to be research integrity stewards and to act as informal 'first responders' to researchers with research integrity questions, in order to guarantee that researchers have access to low-threshold counseling.**
2. Ensure that the volunteers are sufficiently trained in research integrity, although they do not need to have undergone official training specifically targeted at counselors.
3. Harmonize the work of data stewards and RI stewards

*Explanation:* *This item was addressed in both sets of workshops and the SoRs. In the second set of workshops, one participant explained that volunteers also need sufficient training in RI, while others asked for more coherence between the work of data stewards and RI stewards (items 1a and 1b).*

1. **Set clear roles and responsibilities for different bodies/persons involved in counseling & advice**
2. Communicate clearly what the legal responsibilities of each body/role are (e.g. reporting on cases of misconduct)
3. Do not overburden research integrity staff with too many roles (e.g. teaching and handling cases)

*Explanation:* *To prevent RI staff from becoming overburdened and to make it clear and transparent what their different roles and responsibilities are, participants in the second set of workshops suggested to add item 5 to the guideline.*

1. **Ensure that the counselors and research integrity stewards are visible, approachable and easy to find.**
2. Provide information and contact details of counselors and research integrity stewards on the institutional website.
3. Balance visibility with secrecy: Ensure that those approaching the research integrity counselors and stewards can do so without being noticed

*Explanation:* *There was agreement in both sets of workshops on the importance of item 6. Some participants in both workshops brought up item 6a, while one participant in the second workshop raised the issue of balancing visibility with secrecy (item 6b).*

1. **Provide researchers with resources they can consult to become informed and prepare for counseling or advice sessions.**
2. Refer researchers to a European level online helpdesk containing general information on research integrity.

*Explanation:* *In the first set of workshops, participants mentioned that institutions should provide researchers with an online helpdesk which answers simple questions. However, in the second set of workshops, participants were concerned that this would not be feasible for each institution since it would require significant amount of resources. Additionally, some participants in the second set of workshops expressed that simple questions do not exist, as all RI questions they have experienced are context specific and complex. These participants suggested that rather than providing researchers with an institutional helpdesk to address in case of questions, institutions should refer researchers to existing resources that can help them prepare for counseling sessions so that they come to sessions more prepared. On the other hand, a few participants suggested that a helpdesk would be very valuable for ‘simple questions’, but on a European level rather than an institutional level. We have reformulated this item now to include both perspectives (referral to existing resources and to a European level helpdesk).*

1. **Have a strong institutional commitment towards providing RI support.**
2. Include research integrity/ethics as a central aim of the institution
3. Mandate the implementation of the guideline
4. Hold open forums with researchers to explore their needs
5. Allocate sufficient resources and time to counselors, both reactively and proactively.

*Explanation:* *Item 8 is more of an implementation issue for the guideline, rather than a point directly related to RI counseling and advice. However, it was mentioned as a point to include in the guideline in both the first and second set of workshops, since many participants exclaimed that without top down support, the guideline would not work. Items 8a- 8c were additions made in the second set of workshops, to help make the overall item more concrete. Item 8d was raised in the first set of workshops.*

1. **Include counselors & support staff in policy and education, so that counseling can improve policy and education and vice versa.**
2. Co-create institutional policies together with the counselors and support staff
3. Counselors should report on the types of cases they receive to use for education and policy

*Explanation:* *There was agreement about the importance of item 9 in both sets of workshops, since participants mentioned that counseling, policy and education are interrelated and counselors can play a role in helping to align these. However, based on how it is interpreted, this point could be seen to clash with item 5b (not overburdening counselors) in the guideline. Items 9a and 9b were additions made in the second set of workshops to make the overall item more concrete.*

1. **Offer people in support roles the possibility to progress in their career, for instance by involving them in executive decisions of the institution**

*Explanation: Item 10 is more of an implementation issue for the guideline, rather than a point directly related to RI counseling and advice**. Some participants in the first set of workshops emphasized that to ensure good quality counseling, institutions should ensure that counselor are able to climb the career ladder. However, some participants in the second set of workshops questioned the feasibility of this item as it would require a significant budget and resources. A suggestion was made by one of these participants to deal with this feasibility issue by increasing the decision making weight of the counselors, rather than necessarily creating new positions for them. The current formulation of item 10 takes is an attempt to merge these important considerations.*

| **Best practice examples:**   - Ghent university trust point where confidential counselors and RI officers meet with researchers to discuss things |
| --- |

*Additional remarks: The best practice examples above were mentioned by participants in the second set of workshops. There are likely many other best practice examples available which can be added here.*

*Participants also mentioned some implementation considerations for these guidelines in the second set of workshops including that:*

- *Support from the executive board is needed for the implementation of this guideline*
- *Despite the importance of the previous point, to ensure that researchers make use of counseling & advice services offered at the institution, counseling & advice should not just be seen as an extension of the executive board but rather as something that meets the needs of researchers.*
- *In some countries, confidential counselors have a legal duty to report on misconduct cases*
- *RI officers do not have the power to influence many of the items in this guideline (e.g. allowing people to climb the career ladder)*
- *To help implementation, it would be helpful to co-create the institutional policy on counseling and advice together with the community using a bottom up approach*
- *Budget constraints are important for this guideline.*
- *COPE might be interested in helping SOPs4RI develop a European level RI helpdesk.*
