# Supplementary material for: Co-creating Research Integrity Education Guidelines for Research Institutions
Source: Sci Eng Ethics. 2023 Jul 20;29(4):28. doi: 10.1007/s11948-023-00444-2 (PMC10359202; doi:10.1007/s11948-023-00444-2)
Supplement: Supplementary file 4 — Supplementary file4 (DOCX 57 KB) [file 11948_2023_444_MOESM4_ESM.docx]

# Guidelines for research institutions on the **research integrity education of bachelor, master and PhD students**

## Guidelines for research institutions on the **research integrity education of bachelor, master and PhD students**

Research integrity is about conducting high quality research, in accordance with high ethical and professional standards. Research integrity is crucial for the production of trustworthy knowledge. Research institutions have a responsibility to guide and support researchers in conducting research with integrity. One of the key research integrity responsibilities of research institutions is providing education and training in research integrity.

Education and training are needed to raise awareness about research integrity and provide researchers with the needed tools to promote responsible research practices. Research integrity education offered to bachelor, master and PhD students ensures that students learn about responsible research practices at the start of their research trajectory.

This document provides guidance to research institutions on what to include in their research integrity education strategy for bachelor, master and PhD students. We first provide a one page overview of the all the key guideline recommendations. In the subsequent pages, each key recommendation is followed by more detailed guidance and best practice examples to help research institutions bring the recommendations into practice.

The guideline provides information relevant for research officers, trainers, managers, and coordinators, as well as deans, rectors and other institutional leaders. Given the broad diversity that exists among research institutions, it is possible that some recommendations are not applicable in all research settings. For this reason, the guideline should not be seen as a ‘one-size-fits-all’, but rather as a tool that can be used flexibly and adapted to meet institutions’ specific needs.

**Please note:**

• We use the term research integrity **‘education’** to refer to all approaches used to develop understanding, skills, appreciation for, and knowledge about research integrity.

• When we discuss **‘training’**, we refer to specific formal instructional events used for research integrity education, such as courses and workshops.

## Guidelines for research institutions on the **research integrity education of bachelor, master and PhD students**

**Key recommendations:**

1. Integrate into bachelor and master curriculum
2. Deliver mandatory course for PhD students
3. Provide follow-up courses for PhD students
4. Enable informal research integrity discussions
5. Provide train-the-trainer education
6. Use diverse learning environments
7. Focus on concrete experiences
8. Motivate and reward
9. Evaluate

### Integrate into bachelor and master curriculum

Integrate mandatory research integrity education into the bachelor and master curriculum

Starting research integrity education as early as possible in the academic curriculum ensures that students learn responsible research behaviors as they are being taught about research.

1. Set a minimum number of contact hours to dedicate to research integrity throughout the curriculum
2. Integrate research integrity education into the introduction to the study curriculum
3. Integrate research integrity education into the thesis research process

**Best practice examples**

**Example 1**: [Path2Integrity learning materials](https://www.path2integrity.eu/ri-materials)

### Deliver mandatory course for PhD students

Deliver a mandatory research integrity course at the start of the PhD trajectory

A mandatory research integrity course ensures that all PhD students are familiarized with research integrity and empowered to engage in responsible research practices.

1. Provide this research integrity training as a complete course, with a minimum number of contact hours and ECTs
2. Inform students about research integrity principles, policies and norms
3. Stimulate students to share and discuss potential differences in their understanding and application of research integrity norms
4. During the course, stimulate students to discuss potential research integrity challenges as well as ways of dealing with them
5. Organize interactions between PhD students and more senior researchers about research integrity as part of the course
6. Consider involving representatives from multiple disciplines. For instance, a faculty could decide to include both biologists and chemists in one course

**Best practice examples**

**Example 1**: [‘Research ethics for human science’ at Stockholm University](https://www.su.se/department-of-philosophy/education/courses-and-programmes/research-ethics-for-human-science-1.523153?eventopenforinternationalstudents=true&q=&xpanded=)

**Example 2**: [Research integrity training at Nanyang Technological University Singapore](https://www.ntu.edu.sg/research/training-on-research-integrity)

**Example 3**: ‘[Science in Action’ at Pompeu Fabra University (UPF) in Barcelona](https://www.upf.edu/web/phd-biomedicine/science-in-action)

**Example 4**: [‘Responsible Conduct of Research: Integrity in Academic Publishing’ course at Utrecht University](https://www.uu.nl/en/events/responsible-conduct-of-research-integrity-in-academic-publishing-online)

### Provide follow-up courses for PhD students

Provide PhD students with follow-up elective courses on research integrity

As PhD students progress with their research, they will uncover new research integrity questions and challenges. Follow-up resources and research integrity courses on discipline-specific topics can equip students to address new challenges responsibly.

1. Set a minimum requirement about how often students are to follow a discipline-specific elective research integrity course
2. Provide students with access to educational resources on research integrity, such as online training and online accessible materials like codes of conduct and relevant guidelines.

**Best practice examples**

**Example 1**: [‘Research ethics for human science’ at Stockholm University](https://www.su.se/department-of-philosophy/education/courses-and-programmes/research-ethics-for-human-science-1.523153?eventopenforinternationalstudents=true&q=&xpanded=)

**Example 2**: [‘Research data management’ at Vrije Universiteit Amsterdam](https://libguides.vu.nl/rdm)

### Enable informal research integrity discussions

Organize opportunities to discuss research integrity informally

A good research culture entails the possibility for researchers to openly discuss concerns and challenges, and serves as a basis for successful research integrity education.

1. Develop policies for building a responsible research environment, as a prerequisite for open discussion during research integrity education (see our detailed guidelines on community building, skills training, diversity and inclusion, and managing pressure) [anonymized links]
2. Provide concrete suggestions and tools during research integrity training on how researchers can collaborate responsibly with colleagues and supervisors
3. Stimulate faculties and departments to organize a minimum number of informal events each year to discuss research integrity challenges and solutions

- Involve researchers across all seniority levels
- Involve representatives from multiple disciplines

**Best practice examples**

**Example 1**: [‘Met de billen bloot’- Alzheimer Center, Amsterdam UMC](https://embassy.science/wiki/Theme:A12b4bab-b331-46d1-93e0-dc9e9c5453cd)

### Provide train-the-trainer education

Provide train-the-trainer education and basic qualifications for research integrity trainers

Train-the-trainer education provides research integrity trainers with the tools and skills necessary to teach about research integrity. Train-the-trainer education ensures that research integrity trainers are qualified and enthusiastic.

1. Provide train-the-trainer education and qualifications for research integrity trainers, focusing on the basics of research integrity and didactic skills
2. Provide additional topic-specific training and qualifications for trainers of elective discipline-specific research integrity courses (for instance data management training for data management curators)
3. Where necessary, collaborate with trainers or training programs from other institutions to deliver quality research integrity training

**Best practice examples**

**Example 1**: [VIRT2UE training program](https://embassy.science/wiki/Training)

### Use diverse learning environments

Use diverse learning environments, combining online and in-person elements in research integrity education

Diverse learning environments allow students to benefit from the advantages of online and in-person training approaches. Online training can be more efficient for informing students about research integrity basics, and allows students to turn back to training materials and form online support groups. In-person training is suitable for joint discussion of and reflection on the material covered in the online training.

1. Use online training programs to inform students about principles, policies and norms
2. Ensure that students are able to turn back to the online training material at later timepoints and inform students accordingly
3. Use in-person training to stimulate discussion and reflection among students in class
4. Provide students with the means to organize peer support groups and encourage them to maintain contact with their research integrity training peers

**Best practice examples**

**Example 1**: [Epigeum course on research integrity](https://www.epigeum.com/courses/research/research-integrity-uk-edition/)

**Example 2**: [VIRT2UE training program](https://embassy.science/wiki/Training)

**Example 3**: ‘[Science in Action’ at Pompeu Fabra University (UPF) in Barcelona](https://www.upf.edu/web/phd-biomedicine/science-in-action)

**Example 4**: [‘Mind the gap’ –](https://www.kuleuven.be/english/research/integrity/training/mindthegap) Flemish universities

### Focus on concrete experiences

Focus on students’ actual experiences with research rather than merely addressing theory in research integrity education

Focusing on the concrete needs of researchers in their daily practice, rather than merely addressing theory, makes research integrity education appealing, useful and relevant to students. Any research integrity principles, policies or norms taught should be connected to actual research practice.

1. Integrate research integrity principles, policies and standards with discussions of the daily practice of research
2. Discuss case studies and real life examples during research integrity education events
3. Tailor research integrity educational content to the research needs of the target group
   1. Consult with potential participants on what to cover during educational events and update the event based on participants’ needs in practice

**Best practice example**

**Example 1**: [VIRT2UE training program](https://embassy.science/wiki/Training)

### Motivate and reward

Motivate and reward students to actively take part in research integrity education

Motivations and rewards help students see the value and importance of research integrity and foster active engagement with research integrity education.

1. Communicate the purpose and value of research integrity education
2. Frame research integrity training as an opportunity to reflect on how to improve research, rather than an attempt to merely tell students what to do or focus on research misconduct
3. Provide students with a tangible reward after completion of training, such as a digital badges or free meals
4. When possible, consult with students about what rewards and incentives motivate them to engage actively with research integrity education, and tailor these accordingly

### Evaluate

Evaluate educational programs

Evaluations of educational programs provide valuable information to research integrity trainers and institutions on how to improve and further develop research integrity education.

1. Following each research integrity educational event, conduct an evaluation of the event
2. Gather subjective data such as trainees’ perceptions of course usefulness
3. Gather objective data, such as the number of participants enrolled in elective courses
4. Review the evaluation information when organizing the next educational event, to continuously update and improve research integrity education

**Best practice examples**

**Example 1:** [Consider](https://embassy.science/wiki/Training) Kirkpatrick's’ Model for evaluating events

**Example 2:** [Consider measuring integrity indicators at the institution](https://www.nature.com/articles/d41586-021-03493-4)

## Guideline development process

These guidelines are based on empirical work done by the SOPs4RI consortium. We identified available recommendations on the topic, as well as gaps and lacunas using two scoping reviews on best practices for research integrity promotion [1] and the implementation factors related to research integrity [2]; 23 interviews with research integrity experts [3]; a Delphi consensus-study with 68 research policy makers and research leaders across Europe [4]; and 30 focus groups with researchers and other research stakeholders from different disciplines and countries in Europe [5-6]. Following this, we organized 4 co-creation workshops with various research stakeholders to draft the guidelines, with the intention to produce a wide range of practical ideas for the guidelines taking into account users’ needs [7-8]. To revise the guidelines, we worked in a small working group with the aim to prioritize, reorganize and optimize the guideline elements.

Co-creators

Removed for anonymization

Guideline revision working group members

Removed for anonymization

Expert advisors

Removed for anonymization

SOPs4RI guideline development team

Removed for anonymization

**References**

Removed for anonymization
