# Supplementary material for: Co-creating Research Integrity Education Guidelines for Research Institutions
Source: Sci Eng Ethics. 2023 Jul 20;29(4):28. doi: 10.1007/s11948-023-00444-2 (PMC10359202; doi:10.1007/s11948-023-00444-2)
Supplement: Supplementary file 5 — Supplementary file5 (DOCX 56 KB) [file 11948_2023_444_MOESM5_ESM.docx]

# Guidelines for research institutions on the **research integrity education of post-doctorate and senior researchers**

## Guidelines for research institutions on the **research integrity education of post-doctorate and senior researchers**

Research integrity is about conducting high quality research, in accordance with high ethical and professional standards. Research integrity is crucial for the production of trustworthy knowledge. Research institutions have a responsibility to guide and support researchers in conducting research with integrity. One of the key research integrity responsibilities of research institutions is providing education and training in research integrity.

Education and training are needed to raise awareness about research integrity and provide researchers with the needed tools to promote responsible research practices. Research integrity education offered to post-doctorate and senior researchers ensures awareness about research integrity among researchers across seniority levels, and helps researchers to stay up to date with the latest developments in research regulations and innovations.

This document provide guidance to research institutions on what to include in their research integrity education strategy for post-doctorate and senior researchers. We first provide a one page overview of the all the key guideline recommendations. In the subsequent pages, each key recommendation is followed by more detailed guidance and best practice examples to help research institutions bring the recommendations into practice.

The guideline provides information relevant for research officers, trainers, managers, and coordinators, as well as deans, rectors and other institutional leaders. Given the broad diversity that exists among research institutions, it is possible that some recommendations are not applicable in all research settings. For this reason, the guideline should not be seen as a ‘one-size-fits-all’, but rather as a tool that can be used flexibly and adapted to meet institutions’ specific needs.

**Please note:**

- We use the term research integrity **‘education’** to refer to all approaches used to develop understanding, skills, appreciation for, and knowledge about research integrity.
- When we discuss **‘training’**, we refer to specific formal instructional events used for research integrity education, such as courses and workshops.

## Guidelines for research institutions on the **research integrity education of post-doctorate and senior researchers**

**Key recommendations:**

1. Deliver mandatory training for new positions
2. Provide follow-up training
3. Involve seniors in the training of students and juniors
4. Enable informal research integrity discussions
5. Provide train-the trainer education
6. Use diverse learning environments
7. Tailor education to researcher needs
8. Motivate and reward
9. Evaluate

### Deliver mandatory training for new positions

Deliver mandatory training about research integrity for researchers starting new positions

Mandatory training for those starting new positions ensures that researchers have the awareness and skills necessary to conduct their research and fulfil their roles responsibly from the outset.

1. Provide research integrity induction training as a smaller workshop rather than a complete course
2. Include training as part of introduction package for new employees at the institution
3. Include training as part of introduction of employees starting a new position in the same institution, for instance those being promoted as new supervisors or professors
4. Inform researchers about research integrity principles, policies and norms
5. Address the specific responsibilities and skills required for the new position. For instance, training for new supervisors should address supervision skills.
6. Stimulate researchers to discuss research integrity challenges as well as ways of dealing with them
7. Stimulate researchers to share and discuss potential differences in their understanding and application of research integrity norms
8. If post-doctorate researchers have not yet followed a PhD level research integrity training, stimulate them to follow a PhD research integrity course as well

**Best practice examples**

**Example 1**: [Training at University College London](https://www.ucl.ac.uk/research/integrity/research-integrity-training-framework)

**Example 2**: [‘I-Supervise’ by KU Leuven](https://admin.kuleuven.be/personeel/english/trainings/isupervise-masterclass-for-supervisors/isupervise-masterclass-for-supervisors)

**Example 3**: ‘Superb supervision’ at Amsterdam University Medical Centers for [junior](https://www.vumc.nl/educatie/onze-opleidingen/opleidingsdetail/superb-supervision-junior-mentoring-your-phd-candidate-towards-responsible-conduct-of-research.htm) and [senior](https://www.vumc.nl/educatie/onze-opleidingen/opleidingsdetail/superb-supervision-senior-a-course-for-senior-phd-supervisors.htm) supervisors

### Provide follow-up training

Provide researchers with follow-up specialized training on research integrity

Follow-up training focusing on specific research integrity topics – for example training on integrity challenges faced during data management — supports researchers in keeping up with the latest research regulations and policies.

1. Provide follow-up training as smaller workshops rather than complete courses.
2. Set a minimum requirement about how often researchers are to follow a discipline-specific follow-up research integrity training
3. Whenever there are changes to research regulations and policies, provide researchers with educational resources to update them, such as online training and online accessible materials like codes of conduct and relevant guidelines

**Best practice examples**

**Example 1**: [‘Research data management’ at Vrije Universiteit Amsterdam](https://libguides.vu.nl/rdm)

### Involve seniors in the training of students and juniors

Involve senior researchers in the research integrity training of students and junior researchers

Interaction between students, junior and senior researchers about research integrity can help researchers to learn from each other and commit more strongly to research integrity. There are numerous ways to organize such an interaction, so as to motivate juniors and seniors to engage in responsible research practices.

1. Stimulate students and junior researchers to reflect on research integrity together with their supervisors, as part of their research integrity training assignments
2. Invite senior researchers to share experiences, examples, and challenges relating to research integrity as part of the research integrity training of students and junior researchers.

### Enable informal research integrity discussions

Organize opportunities to discuss research integrity informally

An open and inclusive research culture entails the possibility for researchers to openly discuss concerns and challenges and serves as a basis for successful research integrity education.

1. Develop policies for building a responsible research environment, as a prerequisite for open discussion during research integrity education (see our detailed guidelines on community building, skills training, diversity and inclusion, and managing pressure) [links removed for anonymization]
2. Provide concrete suggestions and tools during research integrity training on how to collaborate responsibly with colleagues, supervisors and supervisees
3. Stimulate faculties and departments to organize a minimum number of informal events a year to discuss research integrity challenges and solutions

- Involve researchers across seniority levels
- Involve representatives from multiple disciplines.

**Best practice examples**

**Example 1**: [‘Met de billen bloot’- Alzheimer Center, Amsterdam UMC](https://embassy.science/wiki/Theme:A12b4bab-b331-46d1-93e0-dc9e9c5453cd)

### Provide train-the-trainer education

Provide train-the-trainer education and basic qualifications for research integrity trainers

Train-the-trainer education provides research integrity trainers with the tools and skills necessary to teach about research integrity. Train-the-trainer education ensures that research integrity trainers are qualified and enthusiastic.

1. Provide train-the-trainer education and qualifications for research integrity trainers, focusing on the basics of research integrity and didactic skills
2. Provide additional topic-specific training and qualifications for trainers of elective discipline-specific research integrity courses (for instance data management training for those training researchers on data management)
3. Where necessary, collaborate with trainers or training programs from other institutions to deliver quality research integrity training

**Best practice examples**

**Example 1**: [VIRT2UE training program](https://embassy.science/wiki/Training)

### Use diverse learning environments

Use diverse learning environments, combining online and in-person elements in research integrity education

Diverse learning environments allow researchers to benefit from the advantages of online and in-person training approaches. Online training can be more efficient for informing researchers about research integrity basics, and allows trainees to keep and return to training materials and to form online support groups. In-person training is suitable for joint discussion of and reflection on the material covered in the online training.

1. Use online training programs to inform trainees about principles, policies and norms
2. Provide trainees the option to reuse to the online training material at later timepoints and inform them accordingly
3. Use in-person training to stimulate discussion and reflection among researchers
4. Provide researchers with the means to organize peer support groups and encourage them to maintain contact with their research integrity training peers

**Best practice examples**

Example 1: [VIRT2UE training program](https://embassy.science/wiki/Training)

### Tailor education to researcher needs

Consult with researchers about their research integrity education needs and tailor education accordingly

Researchers from different seniority levels and disciplines might have different research integrity education needs. A bottom up approach – in which researchers are first consulted to assess what their needs are and the education is then tailored accordingly – ensures that research integrity education is useful and relevant.

1. Decide on a frequency at which an education needs analysis will be conducted in the institution
2. When conducting the education needs analysis, include researchers from different ranks and disciplines in the institution
3. Tailor research integrity education events to adequately address the needs identified for the specific target group
4. If possible, when designing new educational events, plan a consultation meeting with potential participants to obtain their input on how to develop and implement the event

### Motivate and reward

Motivate and reward researchers to actively take part in research integrity education

Research integrity education may be perceived as time consuming and of little priority for researchers. Motivations and rewards help researchers see the value and importance of research integrity and foster active engagement with research integrity education.

1. Reward researchers for their participation in research integrity education and for showing commitment to research integrity in promotions and evaluations
2. Communicate the purpose and value of research integrity education
3. Frame research integrity training as an opportunity to reflect on how to improve research, rather than an attempt to merely tell researchers what to do or focus on research misconduct
4. Where necessary, integrate research integrity training into existing mandatory training about research conduct
5. In case of resistance to training, consider labelling training as ‘Masterclass’ rather than training to make them sound appealing
6. In case of resistance to training, consider not labelling training with normative titles such as ‘research integrity’, but rather use more relatable and neutral terms such as ‘research practices’
7. Highlight the importance of research integrity for the institutional and researcher’s reputation

### Evaluate

Evaluate educational programs

Evaluations of educational programs provide valuable information to research integrity trainers and institutions on how to improve and further develop research integrity education.

1. Following each research integrity training or informal educational event, conduct an evaluation of the training or event
2. Gather subjective data such as trainees’ perceptions of course usefulness
3. Gather objective data, such as the number of participants enrolled in elective courses
4. Review the evaluation information when organizing the next educational event, to continuously update and improve research integrity education

**Best practice examples**

**Example 1:** [Consider](https://embassy.science/wiki/Training) Kirkpatrick's’ Model for evaluating events

**Example 2:** [Consider measuring integrity indicators at the institution](https://www.nature.com/articles/d41586-021-03493-4)

## Guideline development process

These guidelines are based on empirical work done by the SOPs4RI consortium. We identified available recommendations on the topic, as well as gaps and lacunas using two scoping reviews on best practices for research integrity promotion [1] and the implementation factors related to research integrity [2]; 23 interviews with research integrity experts [3]; a Delphi consensus-study with 68 research policy makers and research leaders across Europe [4]; and 30 focus groups with researchers and other research stakeholders from different disciplines and countries in Europe [5-6]. Following this, we organized 4 co-creation workshops with various research stakeholders to draft the guidelines, with the intention to produce a wide range of practical ideas for the guidelines taking into account users’ needs [7-8]. To revise the guidelines, we worked in a small working group with the aim to prioritize, reorganize and optimize the guideline elements.

Co-creators

16 co-creators participated in creating these guidelines. Among those, the following consented to be acknowledged:

Removed for anonymization

Guideline revision working group members

Removed for anonymization

Expert advisors

Removed for anonymization

SOPs4RI guideline development team

Removed for anonymization

**References**

## Removed for anonymization
