# Supplementary material for: Co-creating Research Integrity Education Guidelines for Research Institutions
Source: Sci Eng Ethics. 2023 Jul 20;29(4):28. doi: 10.1007/s11948-023-00444-2 (PMC10359202; doi:10.1007/s11948-023-00444-2)
Supplement: Supplementary file 6 — Supplementary file6 (DOCX 49 KB) [file 11948_2023_444_MOESM6_ESM.docx]

# Guidelines for research institutions on the **research integrity education of institutional research integrity stakeholders**

## Guidelines for research institutions on the **research integrity education of institutional research integrity stakeholders**

Research integrity is about conducting high quality research, in accordance with high ethical and professional standards. Research integrity is crucial for the production of trustworthy knowledge. Research institutions have a responsibility to guide and support researchers in conducting research with integrity. One of the key research integrity responsibilities of research institutions is providing education and training in research integrity.

Education and training are needed to raise awareness about research integrity and provide stakeholders with the needed tools to promote responsible research practices. Not only researchers, but also other research integrity stakeholder can benefit from research integrity education. Research integrity education can equip various research integrity stakeholders to adequately support researchers in engaging in responsible research practices.

This document provides guidance to research institutions on what to include in their research integrity education strategy for institutional research integrity stakeholders who are not directly involved in conducting research. We first provide a one page overview of the all the key guideline recommendations. In the subsequent pages, each key recommendation is followed by more detailed guidance and best practice examples to help research institutions bring the recommendations into practice.

The guideline provides information relevant for research officers, trainers, managers, and coordinators, as well as deans, rectors and other institutional leaders. Given the broad diversity that exists among research institutions, it is possible that some recommendations are not applicable in all research settings. For this reason, the guideline should not be seen as a ‘one-size-fits-all’, but rather as a tool that can be used flexibly and adapted to meet institutions’ specific needs.

**Please note:**

- We use the term research integrity **‘education’** to refer to all approaches used to develop understanding, skills, appreciation for, and knowledge about research integrity.
- When we discuss **‘training’**, we refer to specific formal instructional events used for research integrity education, such as courses and workshops.

## Guidelines for research institutions on the **research integrity education of institutional research integrity stakeholders**

**Key recommendations:**

1. Provide basic training
2. Organize coming together events
3. Provide train-the-trainer education
4. Organize follow-up education
5. Provide peer-to-peer learning opportunities
6. Motivate and reward
7. Evaluate

### Provide basic training

Provide institutional research integrity stakeholders who are not performing research with basic research integrity training

*Educating all involved in research about research integrity contributes towards a culture of research integrity. Educating various stakeholders, also those who do not directly conduct research, ensures that various stakeholders are sufficiently informed to support researchers to engage in responsible research practice.*

1. During the training, inform stakeholders about research integrity principles, policies and norms
2. During the training, discuss disciplinary considerations in the application of the principles, policies and norms
3. During the training, inform stakeholders about their responsibilities in supporting researchers with research integrity

### Organize coming together events

Organize events where research integrity stakeholders come together to ask questions, exchange experiences and discuss how to work together on research integrity

Bringing institutional research integrity stakeholders together to share experiences and questions helps them to learn from each other, as well as to work better together in supporting researchers with responsible research practices.

1. Include research integrity committee members, data management personnel, research integrity trainers, research integrity and ethics researchers, research integrity policy staff, confidential counselors, ombudspersons, research integrity officers, and others involved in research integrity
2. Discuss past and potential research integrity case studies relevant for the institution, in a GDPR compliant manner
3. Discuss researchers’ research integrity support needs
4. Discuss various research integrity stakeholders’ roles and responsibilities in supporting researchers with research integrity
5. Discuss disciplinary considerations in the application of research integrity principles, policies and norms
6. Where possible, organize offline events and use online sessions to supplement offline sessions

### Provide train-the-trainer education

Provide train-the-trainer education and basic qualifications for research integrity trainers

Train-the-trainer education provides research integrity trainers with the tools and skills necessary to teach about research integrity. Train-the-trainer education ensures that research integrity trainers are qualified and enthusiastic.

1. Provide train-the-trainer education and qualifications for research integrity trainers, focusing on the basics of research integrity and didactic skills
2. Provide additional topic-specific training and qualifications for trainers of elective discipline-specific research integrity courses (for instance data management training for data management curators)
3. Where necessary, collaborate with trainers or training programs from other institutions to deliver quality research integrity training

**Best practice example**

**Example 1**: [VIRT2UE training](https://embassy.science/wiki/Guide:Bbe860a3-56a9-45f7-b787-031689729e52)

### Organize follow-up education

Organize follow-up educational events when research integrity policies and regulations change

Follow-up educational events are necessary to ensure that support staff and research integrity personnel remain up-to-date with the most recent policies and regulations on research integrity.

1. Integrate policy and regulation changes into the follow up events
2. Use examples and cases to illustrate new policies and regulations

### Provide peer-to-peer learning opportunities

Provide opportunities for peer-to-peer learning about research integrity

Peer-to-peer learning about research integrity can contribute to strengthening the research integrity culture by ensuring that all research stakeholders in the institution are aware of and committed to research integrity.

1. Develop policies for building a responsible research environment, in which researchers can exchange responsible research practices and challenges with each other (see our detailed guidelines on community building, skills training, diversity and inclusion, and managing pressure) [links removed for anonymization]
2. Provide continuous research integrity education to all students and researchers, in which the importance of research integrity for research is highlighted (see our detailed guidelines on this here) [link removed for anonymization]
3. Provide opportunities and financial support for various research integrity stakeholders to participate in national and international support groups, seminars and workshops about research integrity
4. Support open access institutional research integrity resources to allow research integrity personnel to share resources externally an facilitate peer-to-peer learning.

**Best practice examples**

**Example 1**: [ERION](https://www.earma.org/about/governance/thematic-groups/ethics-and-research-integrity-officer-network-erion/)

**Example 2**: [Recaphe](https://recaphe.eu/)

**Example 3**: [Eurashe](https://www.eurashe.eu/)

**Example 4**: [EURAXESS](https://euraxess.ec.europa.eu/)

**Example 5:** [Research integrity lunches offered by the Netherlands Research Integrity Network](https://www.nrin.nl/calendar)

### Motivate and reward

Motivate and reward various research integrity stakeholders to actively take part in research integrity education

Research integrity stakeholders have many tasks and responsibilities. Motivations and rewards can help ensure their active engagement with research integrity education.

1. Reward engagement of institutional research integrity stakeholders in research integrity education during promotions and evaluations
2. Reward the work of research integrity stakeholders in fostering research integrity during promotions and evaluations
3. Reward researchers who also take on research integrity support roles during promotions and evaluations, for instance researchers who also serve as research integrity trainers, confidential advisors, or ombudspersons

### Evaluate

Evaluate educational programs

Evaluations of educational programs provide valuable information to research integrity trainers and institutions on how to improve and further develop research integrity education.

1. Following each research integrity training or informal educational event, conduct an evaluation of the training or event
2. Gather subjective data, such as trainees’ perceptions of course usefulness
3. Gather objective data, such as the number of participants enrolled in optional training
4. Review the evaluation information when organizing the next educational event, to continuously update and improve research integrity education

**Best practice examples**

**Example 1:** [Consider](https://embassy.science/wiki/Training) Kirkpatrick's’ Model for evaluating events

**Example 2:** [Consider measuring integrity indicators at the institution](https://www.nature.com/articles/d41586-021-03493-4)

## Guideline development process

These guidelines are based on empirical work done by the SOPs4RI consortium. We identified available recommendations on the topic, as well as gaps and lacunas using two scoping reviews on best practices for research integrity promotion [1] and the implementation factors related to research integrity [2]; 23 interviews with research integrity experts [3]; a Delphi consensus-study with 68 research policy makers and research leaders across Europe [4]; and 30 focus groups with researchers and other research stakeholders from different disciplines and countries in Europe [5-6]. Following this, we organized 4 co-creation workshops with various research stakeholders to draft the guidelines, with the intention to produce a wide range of practical ideas for the guidelines taking into account users’ needs [7-8]. To revise the guidelines, we worked in a small working group with the aim to prioritize, reorganize and optimize the guideline elements.

Co-creators

16 co-creators participated in creating these guidelines. Among those, the following consented to be acknowledged:

Removed for anonymization

Guideline revision working group members

Removed for anonymization

Expert advisors

Removed for anonymization

SOPs4RI guideline development team

Removed for anonymization

**References**

Removed for anonymization
