# Supplementary material for: Co-creating Research Integrity Education Guidelines for Research Institutions
Source: Sci Eng Ethics. 2023 Jul 20;29(4):28. doi: 10.1007/s11948-023-00444-2 (PMC10359202; doi:10.1007/s11948-023-00444-2)
Supplement: Supplementary file 7 — Supplementary file7 (DOCX 48 KB) [file 11948_2023_444_MOESM7_ESM.docx]

# Guidelines for research institutions on **continuous research integrity education**

## Guidelines for research institutions on **continuous research integrity education**

Research integrity is about conducting high quality research, in accordance with high ethical and professional standards. Research integrity is crucial for the production of trustworthy knowledge. Research institutions have a responsibility to guide and support researchers in conducting research with integrity. One of the key research integrity responsibilities of research institutions is providing education and training in research integrity. Education and training are needed to raise awareness about research integrity and provide researchers with the needed tools to promote responsible research practices.

Training is an important aspect of research integrity education, but continuous research integrity education requires informal approaches to teaching about research integrity as well. These include teaching and learning about research integrity through responsible supervision, socialization in a responsible research environment, as well as learning by doing.

This document provides guidance to research institutions on providing continuous research integrity education outside of formal training. We first provide a one page overview of the all the key guideline recommendations. In the subsequent pages, each key recommendation is followed by more detailed guidance and best practice examples to help research institutions bring the recommendations into practice.

The guideline provides information relevant for research officers, trainers, managers, and coordinators, as well as deans, rectors and other institutional leaders. Given the broad diversity that exists among research institutions, it is possible that some recommendations are not applicable in all research settings. For this reason, the guideline should not be seen as a ‘one-size-fits-all’, but rather as a tool that can be used flexibly and adapted to meet institutions’ specific needs.

**Please note:**

- We use the term research integrity **‘education’** to refer to all approaches used to develop understanding, skills, appreciation for, and knowledge about research integrity.
- When we discuss **‘training’**, we refer to specific formal instructional events used for research integrity education, such as courses and workshops.

## Guidelines for research institutions on **continuous research integrity education**

**Key recommendations:**

1. Provide educational resources
2. Show institutional commitment
3. Provide advice on day-to-day questions
4. Foster responsible supervision and leadership
5. Build a responsible research environment

### Provide educational resources

Provide researchers with educational research integrity resources to consult when needed

As researchers are conducting research, they will encounter questions and challenges. Providing researchers access to research integrity educational resources supports their education in research integrity.

1. Provide researchers with information on where to find institutional policies and guidelines for research integrity
2. Provide researchers with information on available courses, guidelines and additional resources related to research integrity
3. Refer students to offline or online communities where they can exchange experiences and discuss solutions together with other researchers.

**Best practice example**

**Example 1**: [The Embassy of Good Science](http://www.embassy.science)

**Example 2**: [COPE Resources](https://publicationethics.org/core-practices)

**Example 3**: [Editage resources](https://www.editage.com/insights/)

### Show institutional commitment

Show institutional commitment to prove continuous RI education

Continuous research integrity education requires significant institutional commitment to research integrity, for instance in terms of material and human resources.

1. Include research integrity as one of the central value in the institutional mission and vision statement
2. Allocate resources and time to research integrity training for researchers and staff
3. Explore researchers’ research integrity education needs, for example through forums held every few years
4. Stimulate and support research integrity counselors and support staff to contribute towards the formulation of research integrity cases and questions that can be used for research integrity education

### Provide advice on day-to-day questions

Provide researchers with contact persons who can support continuous research integrity education, by providing low-threshold, disciplinary-specific advice on day-to-day research integrity questions

As researchers are conducting research, they will encounter questions and challenges. Having access to low-threshold advice on day-to-day research integrity questions provides context-specific information and advice to researchers.

1. Provide researchers with contact persons for information about domain specific research integrity issues, for instance research integrity or ethics officers, privacy officers, data stewards, librarians and ethics committee members
2. Recruit volunteer researchers in each faculty to act as informal ‘first responders’ to researchers with day-to-day research integrity questions
3. Provide research integrity education and basic qualifications for all contact persons and ‘first responders’
4. Make ‘first responders’ and contact persons’ information and contact details visible on the institutional or faculty website
5. Inform informal ‘first responders’ about each other’s roles so they can refer researchers to one another when necessary

**Best practice example**

**Example 1**: [Research integrity champions at King’s College London](https://www.kcl.ac.uk/research/support/rgei/research-integrity/research-integrity-champions-and-advisors#:~:text=RIAds%20are%20individuals%20recognised%20for,appropriate%20senior%20professional%20services%20staff.)

### Foster responsible supervision and leadership

Develop policies to foster responsible supervision and leadership

Researchers learn about research practice informally through their supervisors and research leaders. Fostering responsible supervision and leadership supports continuous research integrity education.

1. Inform PhD students about responsible supervision (see our detailed guidelines on this [here](https://osf.io/rvnbt/))
2. Foster responsible supervision (see our detailed guidelines on this [here](https://osf.io/4w89m/))
3. Foster responsible leadership (see our detailed guidelines on this [here](https://osf.io/qbpc9/))

**Best practice example**

**Example 1**: [PhD Charter at KU Leuven](https://www.kuleuven.be/english/research/phd/charter)

### Build a responsible research environment

Develop policies for building a responsible research environment

As researchers are socialized in their research environment, fostering a responsible research environment contributes towards continuous research integrity education.

1. Engage in community building for a responsible research culture (see our detailed guidelines on this here) [anonymized link removed]
2. Manage competition and publication pressure (see our detailed guidelines on this here) [anonymized link removed]
3. Provide adequate education and skills training for researchers (see our detailed guidelines on this here) [anonymized link removed]
4. Develop policies on diversity and inclusion (see our detailed guidelines on this here) [anonymized link removed]

**Best practice example**

**Example 1**: [Towards a responsible research climate in Amsterdam](https://amsterdamresearchclimate.nl/)

**Example 2**: [Research culture at KU Leuven](https://www.kuleuven.be/english/research/integrity/culture)

## Guideline development process

These guidelines are based on empirical work done by the SOPs4RI consortium. We identified available recommendations on the topic, as well as gaps and lacunas using two scoping reviews on best practices for research integrity promotion [1] and the implementation factors related to research integrity [2]; 23 interviews with research integrity experts [3]; a Delphi consensus-study with 68 research policy makers and research leaders across Europe [4]; and 30 focus groups with researchers and other research stakeholders from different disciplines and countries in Europe [5-6]. Following this, we organized 4 co-creation workshops with various research stakeholders to draft the guidelines, with the intention to produce a wide range of practical ideas for the guidelines taking into account users’ needs [7-8]. To revise the guidelines, we worked in a small working group with the aim to prioritize, reorganize and optimize the guideline elements.

Co-creators

16 co-creators participated in creating these guidelines. Among those, the following consented to be acknowledged:

Removed for anonymization

Guideline revision working group members

Removed for anonymization

Expert advisors

Removed for anonymization

SOPs4RI guideline development team

Removed for anonymization

**References**

Removed for anonymization
